# Effects of a High Trans Fatty Acid Diet on Kidney-, Liver-, and Heart-Associated Diseases in a Rabbit Model

**DOI:** 10.3390/metabo14080442

**Published:** 2024-08-08

**Authors:** Hammad Ismail, Zaryab Mubashar, Hajra Khan, Zeenat Naveed, Erum Dilshad, Muhammad Zeeshan Bhatti, Sadaf Anwaar, Samreen Saleem, Sabba Mehmood, Abdur Rahman, Umer Rashid, Dalia Fouad, Michel De Waard, Gaber El-Saber Batiha

**Affiliations:** 1 Department of Biochemistry and Biotechnology, University of Gujrat, Gujrat 50700, Pakistan; 2 Department of Bioinformatics and Biosciences, Faculty of Health and Life Sciences, Capital University of Science and Technology, Islamabad 44000, Pakistan; 3 Department of Biological Sciences, National University of Medical Sciences, Rawalpindi 46000, Pakistan; 4 Department of Biological Sciences, International Islamic University, Islamabad 45500, Pakistan; 5 Department of Nutrition and Lifestyle Medicine, Health Services Academy, Islamabad 44000, Pakistan; 6 Punjab University College of Pharmacy, University of the Punjab, Lahore 54590, Pakistan; 7 Department of Zoology, College of Science, King Saud University, P.O. Box 22452, Riyadh 11495, Saudi Arabia; 8 Smartox Biotechnology, 6 rue des Platanes, F-38120 Saint-Egrève, France; 9 L’institut du thorax, INSERM, CNRS, UNIV NANTES, F-44007 Nantes, France; 10 LabEx Ion Channels, Science & Therapeutics, Université de Nice Sophia-Antipolis, F-06560 Valbonne, France; 11 Department of Pharmacology and Therapeutics, Faculty of Veterinary Medicine, Damanhour University, Damanhour 22511, AlBeheira, Egypt

**Keywords:** trans fatty acids, metabolic associated fatty liver disease, coronary vascular disease, coronary kidney disease, elaidic acid, serum biomarkers

## Abstract

Trans fatty acids are specific unsaturated fats found in processed foods that undergo hydrogenation, leading to hepatic disorders such as metabolic-associated fatty liver disease (MAFLD) and conditions like CVD and CKD. The effects of different food samples containing trans fatty acids (elaidic and oleic acid) on the liver, heart, and kidney through antioxidant enzyme activity were investigated in animal models. Liver function tests (ALT, ALP, AST, and LDH), heart biomarker levels (CPK, TC, HDL, LDL, and triglycerides), and kidney biomarker levels (serum creatinine, blood urea nitrogen, and serum uric acid) were examined in serum of rabbits and the histopathology of liver tissues. Results showed that these biomarkers were more elevated in the Mujahid Ghee group than in the normal control, oleic acid, and Kausar Ghee groups. The concentration of antioxidant markers such as peroxidase, glutathione, catalase, thiobarbituric acid reactive substances, and superoxide dismutase were lower in the Mujahid Ghee group. HPLC showed that Mujahid Ghee had the highest quantified value of elaidic acid among all selected samples. Overall, this study demonstrated that elaidic acid in its purest form aggravated MAFLD in rabbit livers and provoked CVK and CVD.

## 1. Introduction

Trans fats (TFAs) are a type of unsaturated fat broadly utilized in the manufacture of food sources, including margarine, snacks, and shortening in fast foods [1]. Compared to the cis configuration, trans fatty acids are usually not common. The chemical bonding of unsaturated fats involves double carbon bonds (-CHCH-). Because of their cis configuration, naturally unsaturated fats are less firmly packed, exhibiting a U-U-shaped bend. Cis fatty acids usually occur in the form of liquids or oils at room temperature. The plasma and tissue levels of trans fat indicate their uptake during the diet because humans cannot produce new TFAs [2]. The uptake of TFAs during diet comes from two primary sources. Partially hydrogenated vegetable oils (PHVOs) are the major roots for TFA production. The other natural origin of TFAs is bacterial isomerases; these enzymes transform the double bonds of plant polyunsaturated fatty acids in the guts of ruminating animals into trans arrangements [3].

Trans fatty acids have liquefying points near room temperature between cis-unsaturated and saturated forms [4]. The reason is that their double bonds have stringent configurations requiring much less space than the double bonding in the cis form. Their unique melting point is in high demand in food manufacturing because it yields advantageous features to foods such as texture and mouthfeel [5]. The main TFA sources are industrially produced pies, cakes, and cookies; frozen deep-fried items (fast food); and many packaged snacks, popcorn, and margarine [6]. A routine high-fat diet (57% of energy from fat) induces hepatic steatosis and liver harm, which are properties of metabolic-associated fatty liver disease (MAFLD). MAFLD occurs as a result of an accumulation of lipids in the liver; with time, the condition can advance to increase the risk of cirrhosis progression, leading to last-stage liver diseases and the cancer of liver cells [7]. The factors associated with MAFLD are metabolic disorders and related conditions, like diabetes, obesity, and dyslipidemia. MAFLD is becoming a significant general medical issue because of the increasing incidence of obesity and type II diabetes [8]. 

The traditional component of the pathogenesis of MAFLD, both metabolically and histologically, is the collection of triacylglycerol (TAG) in the liver [9]. The plasma non-esterified unsaturated fat (NEFA) pool contributes most of the unsaturated fats that stream to the liver in the fast state [10]. This gives the bulk of the unsaturated fats discharged by the liver to VLDL particles. Insulin resistance is also associated with the dysregulation of fat-determined unsaturated fat in the fasting state. Evidence has shown that in patients with MAFLD, insulin does not suppress the lipolysis of adipose tissue [11]. MAFLD is known to be characterized by the highest levels of alanine aminotransferase (ALT), aspartate aminotransferase (AST), and the invasion of the fatty layer over the liver [12]. There have been significant inter-individual changes in people with chronic liver disease [13]. Due to dietary intake, the concentration of TFAs in cellular triacylglycerols and phospholipids is increased. A dangerously higher triacylglycerol concentration in body tissues, i.e., liver, muscle, and pancreatic beta cells, prompts a defensive reaction that involves the activation of inflammatory processes that promote insulin resistance in adipose tissue [14]. Therefore, TFAs are known to be dangerous factors for coronary vascular disease (CVD), insulin resistance, and frequent weight gain leading to systemic inflammation, which are major characteristics of metabolic syndrome (MetS) [15].

Moreover, MAFLD is the most quickly developing sign of concurrent liver-kidney transplantation [16]. It is not only associated with liver-related mortality and morbidity but also a multisystem disease that influences numerous extrahepatic organ systems, such as the kidneys and cardiovascular system [17,18]. CKD results in various disorders like the deregulation of cardio-metabolic activity and liver-related risks [19]. The literature shows an association between MAFLD and CKDs [20]. There is extensive epidemiological evidence linking MAFLD [21] to subclinical atherosclerosis, leading to a higher incidence of CVD [22]. In addition, treatments that focus on MAFLD might help with both the long-term effects on the liver and the long-term effects on the heart in people with MAFLD [23].

Food intake is sometimes the source of trans fatty acids [24], and these trans fatty acids are the contributing factors to the development of MAFLD [25]. This disease strongly affects the liver and related organs. It is accompanied by the development of a fatty layer on the liver, which makes it a fatty liver and affects its functioning. The consumption of a high trans fatty acids diet could also have disastrous effects because it may disturb the lipid profile and can cause harm to many other vital organs [26]. The fundamental objective of this study was to examine the relationships between MAFLD, CKD, and CVD. In addition, this study aimed to analyze the specific functions of various high-fat diets in the progression of MAFLD. 

## 2. Materials and Methods

### 2.1. Sample Collection

In this research, we screened different industrial food products that contain the most hazardous trans fatty acid, known as elaidic acid. We collected various industrially produced food items, i.e., Mujahid Ghee (brand, Pakistani; batch #608; source, local market; composition, RBD oil (50%), palm oil (30%), cottonseed oil (20%), and vitamins A, D, and E), Kausar Ghee (brand, Pakistani; batch #29; source, local market; composition, palm olien (50%), palm oil (35%), soyabean oil (15%), and vitamins A and D), Chibo UHT Dairy Cream (brand, Pakistani; batch #54; source; local market; cow milk with 20% fat), Dubala Dairy Cream (brand, Pakistani; batch #91; source, local market; composition, milk with 15% fat), cheddar cheese slices (brand, Adam’s; batch #225; source, local market; composition, milk, rennet, and emulsifier), and animal fat (bovine fat from local market’s butcher shop). The calorie charts of all collected samples were checked for trans fatty acids. The pure standards of E4637 elaidic acid, ≥99.0% GC grade, and O1008 oleic acid, ≥99.0% GC grade, were purchased from Sigma-Aldrich (Saint Louis, MO, USA).

### 2.2. FT-IR Analysis of Samples

The presence of elaidic acid and oleic acid was confirmed in all the samples by utilizing a Nicolet S5 FT-IR spectrometer (Thermo Scientific, 168 Third Avenue, Waltham, MA, USA) equipped with a 200 km KCl transfer cell and iD7 ATR to obtain high-quality data according to the manufacturer’s protocol for trans fatty acids [27]. Subsequently, the intensity of the elaidic acid levels in test samples was reduced by incorporating mineral oil (OMS) containing a specific concentration (0.03%) at a ratio of 1:2 to work with the stacking of the transfer cell. Before collecting 200 µm KCl cells, all sample mixtures were heated to 50 °C to avoid any chemical reactions during the investigation. Analytical graphs were collected on the Mid-IR scale from 4000–400 cm^−1^ with a resolution of 4 cm^−1^. A clear peak region, i.e., 990–945 cm^−1^, was designated as trans fatty acid during detection.

### 2.3. Animal Maintenance and Treatment Groups

New Zealand White rabbits (males and females) of 900–1300 g in weight were randomly selected and designated as the control, comparative control, and treatment groups (seven rabbits in each group). The animals were kept in healthy conditions in wooden cages and fed with a standard healthy diet at the Primate Facility of the University of Gujrat (UOG), Gujrat, Pakistan. The rabbits were orally administrated 0.7% saline in the control group, and 6 mL/kg/day of oleic acid and elaidic acid standards and 6 mL/kg/day of Mujahid Ghee and Kausar Ghee samples per their body weight for four weeks, twice a day. In our study, we used three diets: a control diet, an EA diet group, and a Mujahid Ghee diet group. The caloric value of the control diet was maintained at 150 kcal/kg/day. The EA and Mujahid Ghee group were given at 6 mL/kg/day, and the caloric value for this 6 mL was maintained at 50 kcal/kg/day. The remaining 100 kcal/kg/day was provided through the normal healthy diet. We ensured that during the whole experiment, the caloric value should be maintained at 150 kcal/kg/day.

### 2.4. Measuring Physical Parameters

The rabbits were monitored daily for physical parameters, including weight, height, and heart rate. The height and weight of the rabbits were calculated using a height scale and a digital weight scale, respectively. The heart rate was calculated by putting the palm on the lower left side of the chest and feeling for the heart beats, counting for 15 s, and then multiplying it by four.

### 2.5. Dissection, Blood Collection, and Tissue Preparation 

The blood was carefully collected from rabbits using a previously described method [28]. Briefly, a 26-gauge syringe was used to collect blood from the central ear artery of the rabbits after cleaning with 95% *v*/*v* alcohol and o-xylene. For dissection, a bunny burrito was made by wrapping the rabbit in a towel to facilitate an intramuscular injection of alfaxalone that can startle the rabbit. Using 3 mL syringes, about ≤1 mL alfaxalone was administered intramuscularly to the caudal epaxial muscle. After the administration of anesthesia, the rabbits were allowed to rest in a quiet cage for 5–10 min to enable sedation to occur. After that, a previously reported method was used to perform the dissection [29]. The rabbit hearts, kidneys, and livers were collected and separated for further analysis. A small portion of each organ was cut and placed in a formalin vial for tissue histopathology. In contrast, a small portion of the tissue was stored in normal saline for antioxidant quantification. Hematoxylin and eosin (H&E) were used for staining, and the slides of the tissue samples were examined at a magnification of 10× and a resolution of 200 nm.

### 2.6. Biochemical Assays for Liver, Heart, and Kidney

The biochemical analyses for liver functions including serum alkaline phosphatase (ALP), aspartate transaminase (AST), alanine transaminase (ALT), and lactate dehydrogenase (LDH); markers for heart cholesterol (TC), triglycerides (TAG), high-density lipoprotein (HDL), and low-density lipoprotein (LDL); and for kidney, including creatinine functions, blood urea nitrogen, and uric acid were determined spectrophotometrically using commercial diagnostics kits supplied by Sigma-Aldrich^®^. The creatine phosphokinase (CPK/CK) level in the serum was estimated using a CPK ELISA kit^®^.

### 2.7. Antioxidant Markers for Liver and Kidney

Peroxidase (POD) is found in almost all animals and plants, and its activity involves eliminating electron species with the help of H_2_O_2_. POD activity in the liver and kidney homogenates was measured according to the protocols (20). The variation in the absorbance of the reaction solution at 460 nm was observed after 1 min, and then POD activity was estimated with an absorbance change of 0.01 as a unit/min [30]. GSH plays a role in redox signaling, cell proliferation regulation, apoptosis, immune function, free radical scavenging, xenobiotic detoxification, fibrogenesis, and cell proliferation regulation. Reaction with 1, 2-dithio-bis-nitrobenzoic acid revealed reduced glutathione in renal homogenate (DTNB). The developed yellow color was immediately viewed at 412 nm on a SmartSpecTM with a spectrophotometer. It was identified as the μM GSH/g tissue [31]. Thiobarbituric acid reactive substances (TBARSs) were used to estimate lipid peroxidation activity in tissue homogenates. After that, 20% trichloroacetic acid and TBA working agent (5% acetic acid and 20% sodium hydroxide) were added to the sample solution. Mixtures were mixed well in a hot water bath for 15 min, followed by an ice shower treatment for 13 min. The absorbance of the supernatant was read at 540 nm by a microplate reader. It was expressed in nM/min/mg tissue protein [31]. The CAT activity was estimated with a slight alteration to the previously reported method. The reaction mixture contained 2 mL of 55 mM phosphate cradle (pH 4.5), 0.4 mL of 6.0 mM H_2_O_2_, and 0.2 mL of tissue homogenate. After 5 min, the absorbance was determined at 250 nm using a microplate reader and results were presented as U/min. The protein concentration of the tissue homogenates was estimated spectrophotometrically with the Bradford technique at 650 nm [31]. The Banister et al. [32] method was used to determine SOD. The absorbance was measured at 550 nm, and the SOD level was expressed as U/mg protein.

### 2.8. HPLC Analysis

The elaidic acid and oleic acid solutions (1000 mg/L) were prepared by dissolving 100 mg of elaidic acid in 100 mL of n-hexane. The six preparations of elaidic acid and oleic acid with concentrations of 3, 5, 100, 300, 500, and 800 mg/L were prepared from a standard stock regimen by decreasing the use of n-hexane as a diluent. Blood samples were collected in ImuMed glass tubes and centrifuged at 1500× *g* for 15 min at 0 °C to obtain serum. The serum was then kept at −80 °C until analysis. The procedure was performed according to the previously described protocol [33]. The C18 column (4.5 mm × 260 mm internal width, and 6 µm particle size) was used for chromatographic separation, UV exposure was applied at 206 nm, elution was applied with a dispensing speed of 3.0 mL/min, and the supply volume was set to 15 µL. The mobile phase was acetonitrile/water (85:15, *v*/*v*) and contained 0.2% acetic acid. The mobile phase was prepared by mixing 700 mL of acetonitrile with 150 mL of HPLC water and 1.5 mL of acetic acid. The retention times of elaidic acid and oleic acid were 2.5 and 4 min, respectively.

### 2.9. Statistical Analysis

GraphPad Prism Version 8.0 (GraphPad Software, Boston, MA, USA) was used to analyze our results statistically. Dunnett’s multiple comparison test was used for ANOVA. The results were expressed as mean ± standard error of the mean, and *p* < 0.05 was considered to indicate significance. 

## 3. Results and Discussion 

### 3.1. Elaidic Acid in Food Samples

**Figure 1:**
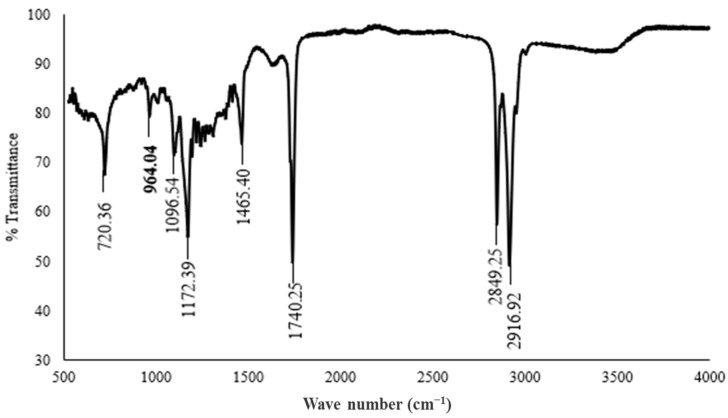
FT-IR spectra of food samples showing the elaidic acid peaks.

Ghee is a traditional dairy product from the Middle East and Asia, especially Pakistan and India, where it is trendy. Nonetheless, ghee use and consumption have increased worldwide in the last few years [34]. To determine the effects of elaidic acid consumption on human nutrition and health, it is necessary to find out the concentration of the trans fatty acids present in locally available samples [35] such as Mujahid ghee. Among the eight samples, only one sample confirmed the presence of elaidic acid. Figure 1 illustrates the FT-IR results; the graph shows the elaidic acid peak at 964 cm^−1^ in the sample of Mujahid Ghee, while the graphs of the rest of the samples are presented in Figure S1. Trans fats have a specified region of 990–945 cm^−1^, and the standard FT-IR peak of elaidic acid is at 966 cm^−1^.

### 3.2. Physical Parameters

**Figure 2:**
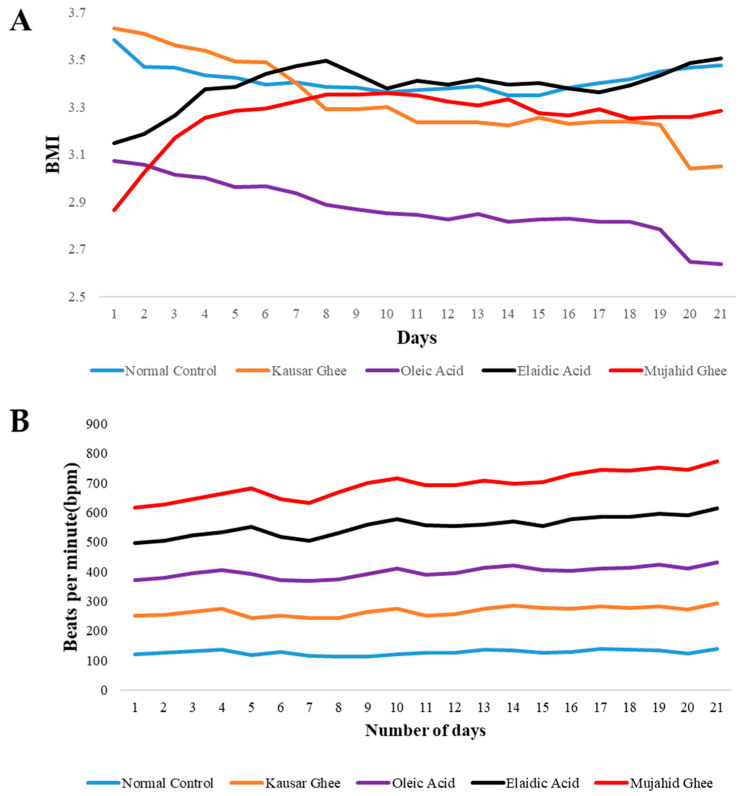
(**A**) Average body mass index (BMI) and (**B**) average heart rate of all groups over 21 days. The values were compared statistically with those of the normal control group at *p* < 0.05.

Figure 2 illustrates the changing trends in the body and mass index (BMI) of the rabbits in conjunction with oleic acid, elaidic acid, Mujahid Ghee, and Kausar Ghee. The BMI progressively increased during the feeding of elaidic acid and Mujahid Ghee, indicating that the rabbits were unhealthy. In the control group, a constant trend was observed, and the BMI of the oleic acid-fed rabbits gradually decreased, indicating that the animals were healthier. Nevertheless, if we examine the Kausar Ghee, we see a variation in its trend. The one-way ANOVA followed by Dunnett’s multiple comparisons test revealed significant differences between the normal control group and all treatment groups. Oleic acid and Mujahid Ghee had significant effects (*p* < 0.001), followed by Kausar Ghee (*p* = 0.001), while elaidic acid had a significant but relatively smaller effect (*p* < 0.046). These findings highlight the potential differential impacts of these substances on the measured outcome and suggest further investigation into the specific mechanisms underlying these effects. The heart rates of the rabbits were examined for 21 days and showed a gradual increasing trend group-wise. The normal control group’s heart rate was calculated as between 120 and 140 beats per minute. In elaidic acid, the heart rate varies from 120 to 180 beats per minute. In the oleic acid group, heart rate was increased from 120 bpm to 140 bpm. In Mujahid Ghee and Kausar Ghee, it varied between 120 bpm to 160 bpm and 130 bpm to 150 bpm, respectively. Therefore, it is noticeable from this research that the high intake of trans fatty acids in the daily routine may be associated with increased heart rates in rabbits of the elaidic acid group and Mujahid Ghee group. Although a rise in heart rate indicates heightened stress levels, it is important to acknowledge that heart rate alone cannot be used to assess stress and anxiety definitively. Further research needs to incorporate other indicators, such as cortisol levels, behavioral observations, and other physiological markers, to offer a more comprehensive assessment of stress and anxiety. It has been reported that an increase in TFA in the diet, without increasing total caloric intake, can cause increased weight and abdominal fat deposition [36]. 

### 3.3. Elaidic Acid in Blood Samples

**Figure 3:**
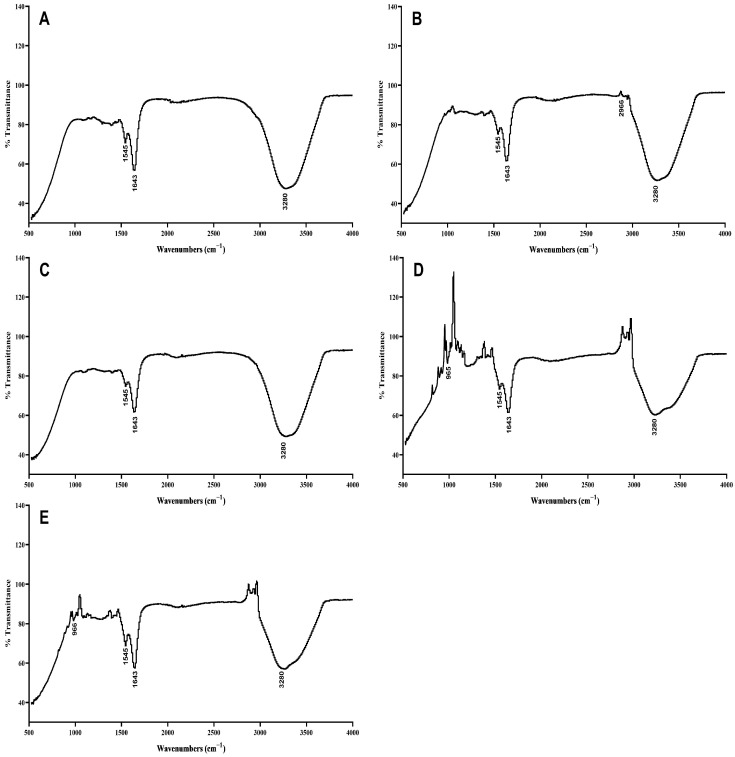
FT-IR spectra of blood samples from (**A**) normal control group, (**B**) oleic acid group, (**C**) Kausar Ghee group, (**D**) Mujahid Ghee group, and (**E**) elaidic acid group.

The following graphs in Figure 3 show the FT-IR results of the blood samples. The graphs of the (d) and (e) samples show peaks corresponding to elaidic acid at 965 cm^−1^ and 966 cm^−1^, respectively, while the remaining graphs do not show any trans fat peaks in the abovementioned range. The absorption peak at 2966 cm^−1^ in graph (b) is much closer to the actual peak at 3006 cm^−1^, as reported previously, which is assigned to the unsaturated C–H groups of cis-olefin, a functional part of oleic acid. The spectra of pure oleic and elaidic acids showed that in the end-line groups of alkyl chains—CH3 and CH2—absorption peaks indicate the extending and bending vibrations of C–H. The region at 3000–2800 cm^−1^ shows extending vibrations, while the region at 1500–1300 cm^−1^ shows bending vibrations. The IR range of elaidic acid displayed in graph (e) corresponds with a formerly detailed IR range of pure elaidic acid. The smallest peak at 3034 cm^−1^ and the largest at 966 cm^−1^ correspond to the extension of C–H bonds and the out-of-plane bending of consecutive double bonds in elaidic acid, which are normal IR range attributes of unadulterated elaidic acid and are missing in the range of oleic acid [37]. According to the literature on infrared bands in human blood, stretching and bending planes are observed in the following regions: 3500–3200 cm^−1^ for water and hydroxyl (O–H stretching), 3285 cm^−1^ for amide A (N–H extending), 2959 cm^−1^ for lipids (asymmetric extending of CH3), 2873 cm^−1^ for lipids (symmetric extending of CH3), 1700–1600 cm^−1^ for amide I (C=O extending), 1560–1500 cm^−1^ for amide II (N–H bending and C–N extending), 1390 cm^−1^ for lipids and proteins (symmetric twisting of CH3), 1239 cm^−1^ for amide III (C–N extending), 1082 cm^−1^ for glucose (C–O extending), and 698 cm^−1^ for amide IV (C–H bending) [27]. It is speculated that the highlighted peaks at 965–975 cm^−1^ could differ between the cis and trans configurations of trans fatty acids [37]. In this study, FTIR confirmed the uptake of elaidic acid by the blood of rabbits who consumed a diet rich in elaidic acid. Our results align with the findings of Mozaffarian et al. [38], who demonstrated that diets high in trans fatty acids, such as elaidic acid, lead to increased levels of these fatty acids in the bloodstream. Similarly, Baer et al. [39] reported that the incorporation of trans fats into the diet results in their detectable presence in blood samples, corroborating our observations.

### 3.4. Determination of Biomarkers in Serum

The effects of trans fatty acids on liver biochemical markers (i.e., ALT, AST, ALP, LDH), heart biochemical markers (i.e., CPK, cholesterol, triglycerides, HDL, and LDL) and kidney biochemical markers (i.e., creatinine level, blood urea nitrogen, and uric acid) were estimated using Sigma-Aldrich kits. The results in Table 1 show that the serum markers were significantly higher in pure elaidic acid, elaidic acid-rich, and Mujahid Ghee diets, compared to the diets of normal control and comparative control groups (oleic acid and Kausar Ghee, respectively). The levels of plasma hepato-specific enzymes such as ALT, AST, ALP, and LDH were significantly increased (*p* < 0.05) in the high-fat-fed rabbits as compared to normal control and comparative control groups (Table 1). Feeding elaidic acid led to significantly higher levels of AST, ALP, and LDH compared to the normal control group (*p* < 0.05). Commonly used hepatic enzymes are ALT and AST, which are used to indicate the initial stage of liver disease. They also play a role in indicating hepatocellular injury and liver [40]. The plasma ALP concentration is sensitive for detecting intrahepatic and extrahepatic bile obstructions [15]. 

LDH is useful for diagnosis or as an indicator for many diseases in the liver and muscles and also for cancer. If we need to confirm that a high level of LDH results from muscular disorders and not from other conditions, it is important to measure the CPK level [41]. CPK levels were more enhanced in groups fed with a high-fat diet (HFD) than in other groups, because of minor myocardial injury. CPK is a better indicator of heart or muscular damage. Therefore, CPK estimation and LDH may serve as suitable diagnostic markers for acute myocardial infarction [41]. 

In our research, the TC, TG, and LDL levels were higher in the TFA-rich fed groups than in the control groups. Meanwhile, the comparative and normal control groups had higher HDL concentrations. The administration of an HFD, an extraordinarily trans isomer carrying an eating routine, results in a critical increase in the absolute cholesterol, triacylglycerol, and phospholipid levels in plasma, which is combined with increased serum LDL-C level and decreased coursing HDL-C, resulting in a model of dietary hyperlipidemia [42,43]. 

By examining kidney biomarkers, it was concluded that serum samples from rabbits treated with elaidic acid and Mujahid Ghee had elevated levels of creatinine, uric acid, and blood urea nitrogen compared to the normal control and comparative control serum samples. Creatinine and urea are acceptable markers of normal kidney function; creatinine is synthesized in the liver and is principally removed from muscle tissue for storage. When filtration does not occur, creatinine levels rise in the body, which ultimately defines the level of damage that has occurred in the kidneys [44]. In the presence of renal disease, the renal ability to excrete creatinine and urea is lost, and the serum creatinine/BUN ratio is elevated. Uric acid is the final product of purine metabolism in humans. The kidney excretes uric acid, and a comparably high uric acid level in serum indicates a reduced glomerular filtration rate (GFR), one of the symptoms of a diseased kidney [45]. Our findings align with those of previous studies, which have shown that diets high in trans fatty acids are associated with impaired kidney function. For instance, studies by Lichtenstein [46] reported elevated levels of creatinine and BUN in subjects consuming high trans fat diets, indicating renal dysfunction. Moreover, the elevated uric acid levels in our study corroborate the findings of Julie et al. [47], who observed that high dietary trans fat intake is linked to reduced GFR and impaired kidney function.

### 3.5. Determination of Antioxidant Enzymes

Oxidation reactions in the body produce free radicals, leading to chain reactions that may damage the organism or the cell. Antioxidants counter the action of these free radicals and protect cells from destruction. When the balance between ROS production and antioxidant defense is lost, oxidative stress, which deregulates cellular functions through a series of events, leads to various pathological conditions. GSH, CAT, and POD constitute a mutually supportive team of defense mechanisms against reactive oxygen species [48]. In the present study, POD, TBARS, CAT, and SOD enzyme activity and GSH protein levels in hepatic and renal tissues were lower in the Mujahid group and the pure elaidic acid group as compared to the normal control group. The overall results for both the liver and kidney are shown in Table 2. It is proposed that not only a high-fat diet but also free radicals induce a fatty liver and a fatty kidney through a related process, which includes the peroxidation of lipids [49]. 

Peroxidase (POD) enzymes are the class of enzymes present in various organisms, and glutathione peroxidase is a peroxidase substrate. Recent experiments have indicated that TFAs decrease the fluidity of the cell membrane. When TFAs are integrated into the membranes of cells, the fluidity of the membrane tends to decline, indicating the improper functioning of the cell. The production of reactive oxygen species is promoted as an effect of improper cell functioning, which indicates the increase in lipid peroxidation in those groups fed with a high-TFA diet [15]. POD activity in the liver and kidney of the elaidic acid and Mujahid Ghee groups was lower than that of the oleic acid and comparative control groups. Glutathione is a tripeptide found throughout the body and is central to oxidative stress management. Livers and kidneys also need an adequate supply of glutathione for normal functioning. TBARS is presumably the most established and perhaps the most broadly utilized test for estimating the end product of lipid peroxidation malondialdehyde, which is a receptive aldehyde delivered by the lipid peroxidation of polyunsaturated fatty acids [50].

Catalase exists in peroxisomes, which comprise two hydrogen peroxide (H_2_O_2_) molecules [51]. SOD protects several tissues against oxidative damage, so SOD acts as an antioxidant defense system [52]. SOD activity in the elaidic acid group was the lowest, and the activity in the Mujahid Ghee group also decreased compared to that in the oleic acid group. Nevertheless, both oleic acid and the comparative control groups also showed significant differences compared to the normal control group. It has been demonstrated in various studies that Mujahid Ghee as a TFA source can induce oxidative stress. TFAs provoke ROS and reduce the functioning of antioxidant enzymes by mostly decreasing GSH levels and SOD activity [15].

### 3.6. HPLC Analysis of Serum

**Figure 4:**
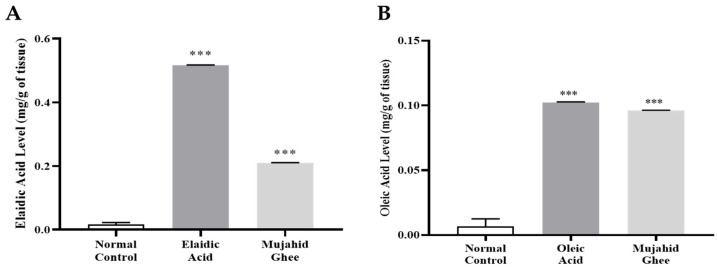
(**A**) Elaidic acid levels in serum. (**B**) Oleic acid levels in serum. Here *** *p* < 0.001 compared with the normal control group.

The quantitative evaluation of elaidic acid in serum was performed using reversed-phase HPLC, the isocratic elution mode, and a diode array detector. All the HPLC graphs are presented in Figure S2. According to the analysis, the retention times for elaidic acid, Mujahid Ghee, and oleic acid were 3.998 min, 4.085 min, and 2.534 min, respectively. These values suggest that the retention time is dependent on the degree of unsaturation. Elaidic acid and Mujahid Ghee are more unsaturated than oleic acid, so they took more time to elute. Furthermore, the results of HPLC analysis of the elaidic acid calibration curve showed that the concentration of the elaidic acid was 0.51 mg/g, which is much higher than the values of 0.23 mg/g of oleic acid and 0.20 mg/g Mujahid Ghee (Figure 4). Similarly, the results of the HPLC analysis of the oleic acid calibration curve showed that the concentration of elaidic acid was 0.16 mg/g, which is also higher than that of 0.10 mg/g of oleic acid and 0.09 mg/g of Mujahid Ghee. Investigations have revealed a straightforward relationship between serum TFAs and the utilization of TFAs. The serum TFA level may reflect the body’s unsaturated fat synthesis, edible fat nature, and fat utilized during significant stretching [53]. Various adverse impacts of trans unsaturated fats collectively, as well as those created by incomplete hydrogenation on CVD, blood lipids, irritation, oxidative pressure, endothelial wellbeing, body weight, insulin affectability, and malignant growth specifically, have been previously confirmed [54]. Various studies have also confirmed that elaidic acid is the key fraction of trans fatty acids taken into the body through the consumption of PHVOs [55].

Our findings are in agreement with previous research showing that diets that are high in trans fatty acids suppress the antioxidant defense system. For example, Dhibi et al. [15] reported that the ingestion of trans fats is correlated with a reduction in the activity of antioxidant enzymes in animal models. In the same way, research conducted by Li et al. [56] has demonstrated that trans lipids can result in the elevated levels of oxidative stress and the deficiencies of critical antioxidant enzymes, such as SOD and CAT, in renal and hepatic tissues. These studies validate our observations of decreased POD, TBARS, CAT, SOD activities, and GSH levels in the trans fat-fed groups, leading to oxidative stress and associated health outcomes.

### 3.7. HPLC Analysis of Organ Tissue

**Figure 5:**
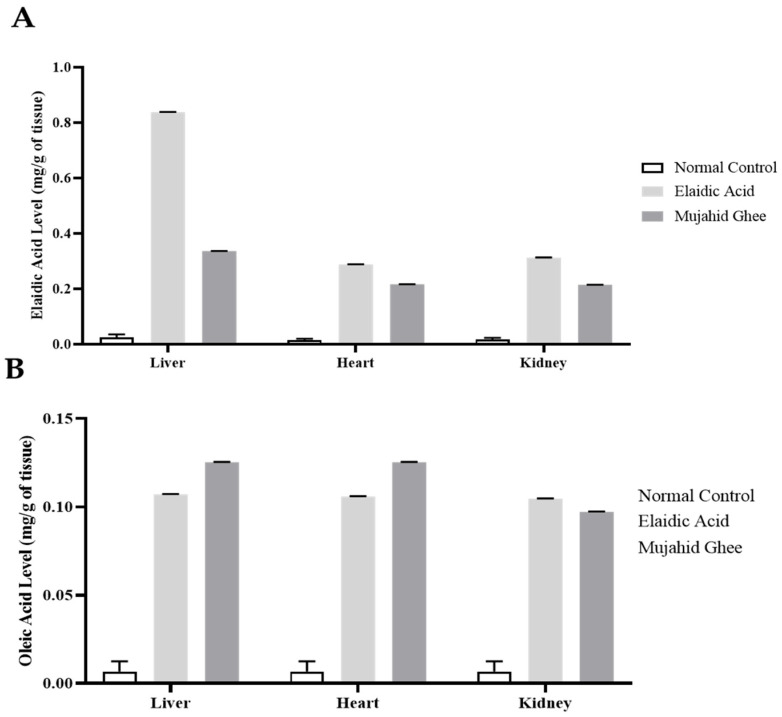
(**A**) Elaidic acid levels in organs. (**B**) Oleic acid levels in organs.

The quantitative evaluation of elaidic acid in the liver, kidney, and heart was carried out using reversed-phase HPLC, and all the HPLC graphs are presented in Figures S3, S4 and S5, respectively. The results of the HPLC analysis of the elaidic acid calibration curve showed that the level of elaidic acid in the liver was higher in the liver as compared to the heart and kidney at 0.83 mg/g, which is greater than 0.25 mg/g of oleic acid and 0.33 mg/g of Mujahid Ghee in liver tissues, as shown in Figure 5. The second highest value of elaidic acid was recorded in the kidney, which was 0.31 mg/g, higher than 0.24 mg/g of Oleic Acid and 0.21 mg/g of Mujahid Ghee. The heart had the lowest concentration, but the significant value of elaidic acid was 0.28 mg/g, which was slightly higher compared to 0.25 mg/g of oleic acid and 0.21 mg/g of Mujahid Ghee. Similarly, the results of HPLC analysis of the oleic acid calibration curve showed that the concentration of elaidic acid in the liver was 0.24 mg/g, which is greater than that of 0.10 mg/g of oleic acid and 0.12 mg/g of Mujahid Ghee, as shown in Figure 5. In the case of the heart, the concentration of elaidic acid was 0.11 mg/g, which is slightly greater than the 0.10 mg/g of oleic acid and 0.09 mg/g of Mujahid Ghee. In contrast, in the kidney, the concentration of elaidic acid was 0.11 mg/g, which is greater than the 0.10 mg/g of oleic acid and 0.09 mg/g of Mujahid Ghee. Several studies have focused on the impact of food sources rich in trans fatty acids on hepatic capacity and oxidative pressure. Oxidative stress is currently recognized to be a significant part of the progression of MAFLD [15]. It is reported that hepatic unsaturated fat might affect the level of liver injury [57]. Dietary fatty acids, mainly unsaturated fatty acids, greatly influence the phospholipid content and fatty acid composition of cellular membranes. Moreover, in another study, trans-octadecenoic isomers were detected in various biopsy specimens collected during open-heart surgery [58]. CKD is a complicated, developing chronic kidney state that can be explained as the irregularity of the kidney structure and is a serious health condition [59]. 

The present research shows that the intake of trans fats provokes liver dysfunction, which causes disturbances in the liver lipid profile that result in MAFLD. MAFLD can indirectly impair many other organs, especially in the kidneys, while TFAs directly impact the heart function. Our results are consistent with prior research that has demonstrated the detrimental effects of trans lipids on liver health. For example, Dhibi et al. [15]] showed that hepatic steatosis and inflammation, which are essential characteristics of MAFLD, are correlated with trans fat consumption. Furthermore, de Zhao et al. [60] demonstrated that trans fats increase liver lipid accumulation and change lipid metabolism, which supports our observations of liver dysfunction in TFA-fed groups.

### 3.8. Histopathology of Organ Tissues

**Figure 6:**
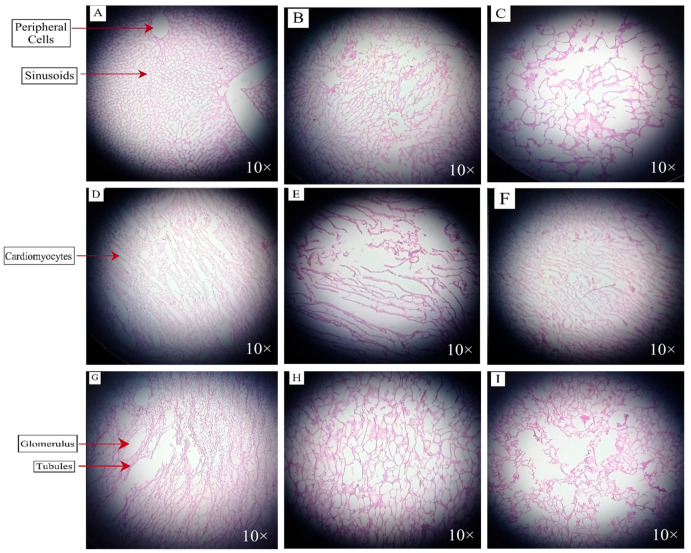
Liver tissues of the (**A**) control group, (**B**) elaidic acid group, (**C**) oleic acid group, heart tissues of (**D**) control group, (**E**) elaidic acid group, and (**F**) oleic acid group; kidney tissues of the (**G**) control group, (**H**) elaidic acid group, and (**I**) oleic acid group.

The dissection of the organs was performed, and during dissection, enlarged livers covered in a fatty layer on the lateral side were observed. The sections prepared from rabbit organs were stained with eosin and hematoxylin. The samples were observed at various resolutions to study the physiological changes in the rabbits of each group. The microscopic images of liver, heart, and kidney samples of different groups are shown in Figure 6. Slides were observed using a light microscope at a magnification of 10×. In normal control liver tissues, a visible pattern of hepatocytes was observed with clear sinusoids and peripheral cells without black spots. In the elaidic acid group, densely populated hepatocytes with unclear sinusoids and some blackish spots were observed. In the oleic acid group, hepatocytes with lysed cell membranes were enlarged, and most of the cells were damaged. These findings suggested that the regular usage of cholesterol/fat in the eating routines for three weeks caused lipid accumulation in the cytoplasm of the hepatocytes, resulting in the ballooning of the liver cells. The livers of the elaidic acid group and oleic acid group seemed pale, delicate, mottled, and fatty, and they were larger than those of the other groups [61].

Vertically packed cardiomyocytes were visible in the normal control heart tissues. In the elaidic acid group, cardiomyocytes were highly degraded and were not visible. In the oleic acid group, clear cardiomyocytes were observed with perfect alignment. The histopathological assessment of the heart revealed the enhanced levels of inflammatory mono-nuclear and hypertrophic heart cells in the elaidic acid group. It also begins within the myocardium as a versatile reaction, and then, at that point, causes free radical activation and chronic inflammatory cell hypertrophy. Myocardial hypertrophy is caused by enhanced mitochondrial oxidative stress as a consequence of a routine high-fat eating routine [62]. 

In the kidney tissues of the normal control group, the tubular overlap was observed with the absence of a clear Bowman’s capsule, and a clear glomerulus was observed. In the elaidic acid group, an expanded glomerulus, cell proliferation in the glomeruli, and the thickening of tubular membranes were observed. A disrupted Bowman’s capsule with overlapping tubules was noticed in the oleic acid group. Figure 6 shows that there is noticeable glomerular hyperfiltration in the initial stages of kidney diseases. It is recommended that the enlargement of the glomerulus after high-fat diet feeding occurs because of an increase in metabolic parameters in obese animals, which leads to glomerular hyperfiltration [63].

### 3.9. Comparison of Oleic Acid and Elaidic Acid

During our research, we compared the impacts of diets rich in trans fatty acids (TFAs) and diets rich in oleic acid on different health indices. Both diets led to an increase in BMI and caused certain metabolic alterations. However, there was a notable difference in the effect on stress indicators and cardiovascular risk between the two diet groups. The consumption of diets rich in trans fatty acids was found to be linked to elevated stress levels and heart rates, indicating a detrimental impact on cardiovascular health and stress [25]. On the other hand, a diet rich in oleic acid, although it led to a weight increase, had a smaller effect on stress markers. This is probably because oleic acid has positive effects on lipid profiles and inflammation [64]. These findings emphasize the need to consider the specific kind of dietary lipids ingested rather than solely focusing on the amount. Although both forms of unsaturated fats can contribute to weight gain, their long-term health consequences vary significantly. Future studies are needed, focusing on the precise processes by which trans fatty acids and oleic acid impact metabolic health and stress. Our analysis concludes that both trans fatty acids and oleic acid can increase body mass index and certain metabolic factors. However, TFAs have negative effects on stress and cardiovascular health. This highlights the importance of dietary guidelines that promote the consumption of healthier fats, such as oleic acid [65].

## 4. Conclusions

Our main objective was to investigate the effects of trans fatty acids on the liver, kidney, and heart to examine changes in biochemical markers and antioxidant enzymes. The levels of biochemical markers were increased, and the levels of antioxidant enzymes decreased, exhibiting a gradual trend in the Mujahid Ghee group compared to those of the oleic acid group. Overall, HPLC and FTIR results illustrated that the quantity of elaidic acid was higher in the Mujahid Ghee than in the other fatty acid samples. Our selected diets, which showed higher values of elaidic acid, had adverse effects on the liver, kidney, and heart of rabbits and were capable of provoking MAFLD. However, further investigation is needed to identify and isolate the specific trans fats that are involved in enhancing the chances of MAFLD. This will further lead to the investigation of the pathways responsible for the production of these TFAs so that these trans fatty acids can be eliminated from the processed foods present in the local markets.

## Figures and Tables

**Table 1:** Results of biomarkers of liver, heart, and kidney present in serum.

Liver					
Biomarker	Normal Control	Kausar Ghee	Oleic Acid	Elaidic Acid	Mujahid Ghee
ALT (IU/L)	47.71 ± 0.87	51.51 ± 0.76 ***	49.4 ± 0.77 **	74.68 ± 0.92 ***	64.67 ± 0.82 ***
AST (IU/L)	44.51 ± 1.42	55.62 ± 0.98 ***	46.77 ± 0.98 **	111.31 ± 1.08 ***	86.72 ± 1.13 ***
ALP (IU/L)	32.84 ± 1.16	47.95 ± 1.08 ***	34.4 ± 1.30 *	79.31 ± 0.76 ***	58.74 ± 1.02 ***
LDH (IU/L)	309.88 ± 1.30	313.74 ± 1.26 ***	311.78 ± 1.09 *	351.81 ± 0.98 ***	332.14 ± 1.40 ***
**Heart**					
CPK (IU/L)	152.34 ± 0.69	160.31 ± 0.67 ***	155.01 ± 0.97 ***	253.87 ± 0.9 ***	210.57 ± 0.87 ***
TC (mg/dL)	48.1 ± 0.77	58.01 ± 0.99 ***	51.31 ± 0.79 ***	78.45 ± 0.74 ***	65.64 ± 0.56 ***
HDL (mg/dL)	24.27 ± 0.55	21.1 ± 0.64 ***	26.31 ± 0.79 ***	16 ± 0.7 ***	18.3 ± 7.2 ***
LDL (mg/dL)	26.81 ± 0.60	34.64 ± 0.82 ***	30.35 ± 0.75 ***	48.35 ± 0.51 ***	45.68 ± 0.78 ***
TAG (mg/dL)	63.28 ± 0.84	70.11 ± 0.72 ***	66.27 ± 0.76 ***	90.47 ± 0.84 ***	82.58 ± 0.46 ***
**Kidney**					
Serum Creatinine (mg/dL)	0.728 ± 0.33	0.82 ± 0.34 ^NS^	1.5 ± 0.40 *	2.97 ± 0.73 ***	2.44 ± 0.67 **
Blood Urea Nitrogen (mg/dL)	17.28 ± 0.89	18.64 ± 1.90 *	19.72 ± 0.94 **	24.12 ± 0.89 ***	20.84 ± 1.05 ***
Serum Uric Acid (mg/dL)	4.77 ± 0.59	5.7 ± 0.8 ^NS^	6.08 ± 0.37 ***	8.22 ± 0.69 ***	8.04 ± 0.74 ***

Here * *p* > 0.05, ** *p* > 0.01, and *** *p* > 0.001. NS is non-significant in the normal control group.

**Table 2:** Results of antioxidant enzymes present in liver and kidney.

Liver					
Enzyme	Normal Control	Kausar Ghee	Oleic Acid	Elaidic Acid	Mujahid Ghee
Total protein (μg/mg tissue)	13.83 ± 0.73	13.65 ± 0.32	14.21 ± 0.58	13.12 ± 0.61	14.30 ± 0.51
POD (U/min)	0.18 ± 0.01	0.16 ± 0.004 ^NS^	0.14 ± 0.02 *	0.12 ± 0.01 **	0.06 ± 0.04 ***
GSH (mM/g tissue)	0.96 ± 0.04	0.89 ± 0.06 ^NS^	0.62 ± 0.24 ***	0.33 ± 0.01 ***	0.36 ± 0.13 ***
TBARS (nM/min/mg tissue)	4.15 ± 0.01	2.34 ± 0.15 ***	3.45 ± 0.18 ***	1.05 ± 0.02 ***	1.56 ± 0.25 ***
CAT (U/min)	2.28 ± 0.92	1.07 ± 1.09 *	0.69 ± 0.56 **	0.52 ± 0.18 ***	0.89 ± 0.87 ***
SOD (U/mg protein)	4.58 ± 0.57	4.05 ± 0.04 ^NS^	3.31 ± 1.07 *	2.08 ± 0.59 ***	3.21 ± 1.33 *
**Kidney**					
Total protein (μg/mg tissue)	13.27 ± 0.64	13.38 ± 0.46	13.49 ± 0.99	12.38 ± 0.0.75	13.48 ± 0.65
POD (U/min)	0.17 ± 0.01	0.13 ± 0.018 *	0.129 ± 0.02 **	0.123 ± 0.02 ***	0.10 ± 0.01 ***
GSH (mM/g tissue)	0.85 ± 0.04	0.59 ± 0.17 *	0.61 ± 0.24 *	0.14 ± 0.08 ***	0.31 ± 0.15 ***
TBARS (nM/min/mg tissue)	3.40 ± 0.31	2.16 ± 0.74 ***	2.63 ± 0.57 *	0.16 ± 0.07 ***	0.93 ± 0.24 ***
CAT (U/min)	2.59 ± 1.39	1.30 ± 0.73 *	0.70 ± 0.42 ***	0.65 ± 0.42 ***	0.79 ± 0.57 ***
SOD (U/mg protein)	4.11 ± 0.50	3.23 ± 0.41 *	2.99 ± 0.45 **	0.73 ± 0.0.47 ***	1.48 ± 0.80 ***

Here * *p* > 0.05, ** *p* > 0.01, and *** *p* > 0.001; NS is non-significant in the normal control group.

## Data Availability

The data presented in this study are available within the article and the Supplementary Materials.

## Supplementary Materials

The following supporting information can be downloaded at: https://www.mdpi.com/article/10.3390/metabo14080442/s1. Figure S1: FT-IR spectrums of food samples: (a) Kausar Ghee; (b) Mujahid Ghee; (c) Blue Band Margarine; (d) Belle Margarine; (e) Chibo UHT Dairy Cream; (f) Dubala Dairy Cream; (g) cheese; (h) animal fat; Figure S2: HPLC analysis of serum: (a) control group; (b) oleic acid group; (c) elaidic acid group; (d) Mujahid Ghee group; (e) Kausar Ghee group; Figure S3: HPLC results of heart tissue: (a) control group; (b) oleic acid group; (c) elaidic acid group; (d) Mujahid Ghee group; (e) Kausar Ghee group; Figure S4: HPLC results of liver tissue: (a) control group; (b) oleic acid group; (c) elaidic acid group; (d) Mujahid Ghee group; (e) Kausar Ghee group; Figure S5: HPLC results of kidney tissue: (a) control group; (b) oleic acid group; (c) elaidic acid group; (d) Mujahid Ghee group; (e) Kausar Ghee group.
