# Supplementary material for: Effects of a High Trans Fatty Acid Diet on Kidney-, Liver-, and Heart-Associated Diseases in a Rabbit Model
Source: Metabolites. 2024 Aug 8;14(8):442. doi: 10.3390/metabo14080442 (PMC11356145; doi:10.3390/metabo14080442)
Supplement: Supplementary file 1 [file metabolites-14-00442-s001.zip › metabolites-3120725-supplementary.pdf]

Supplementary Data

Effects of a High Trans Fatty Acid Diet on Kidney-, Liver-, and Heart-Associated Diseases in a Rabbit Model

a)

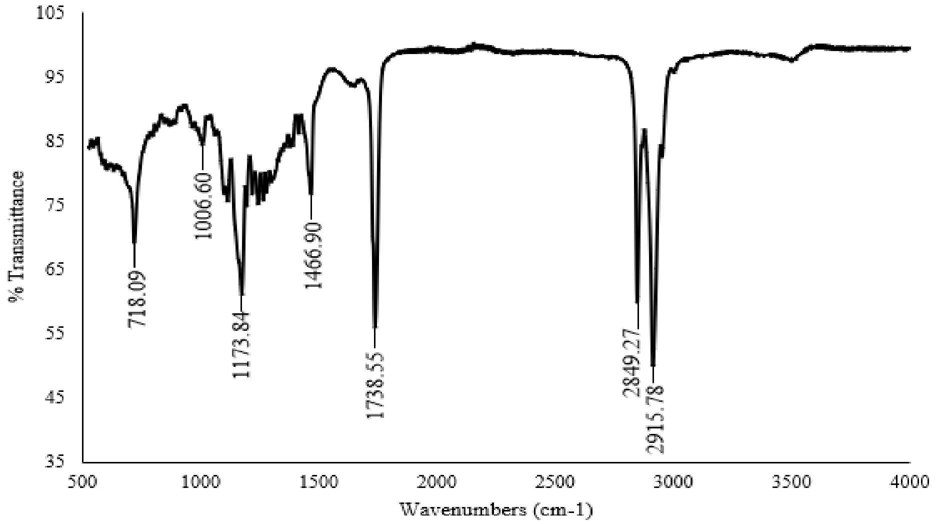

b)

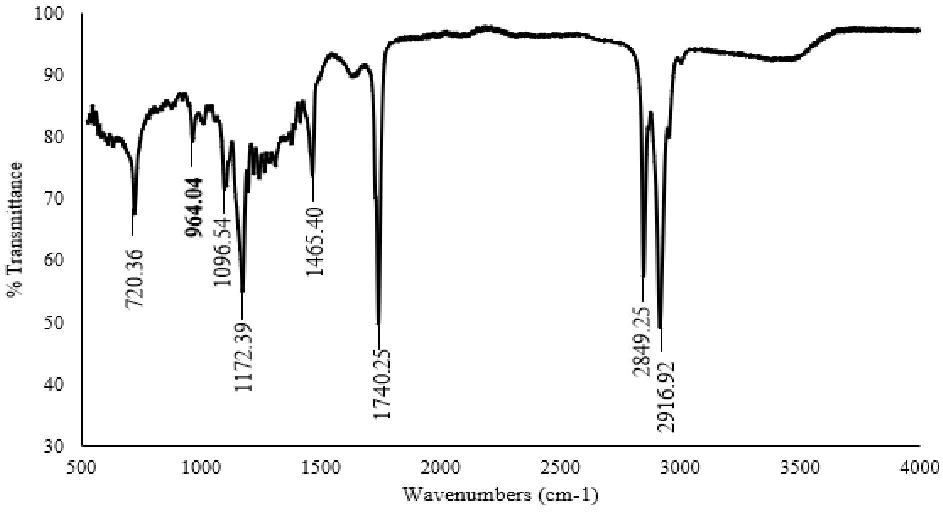

c)

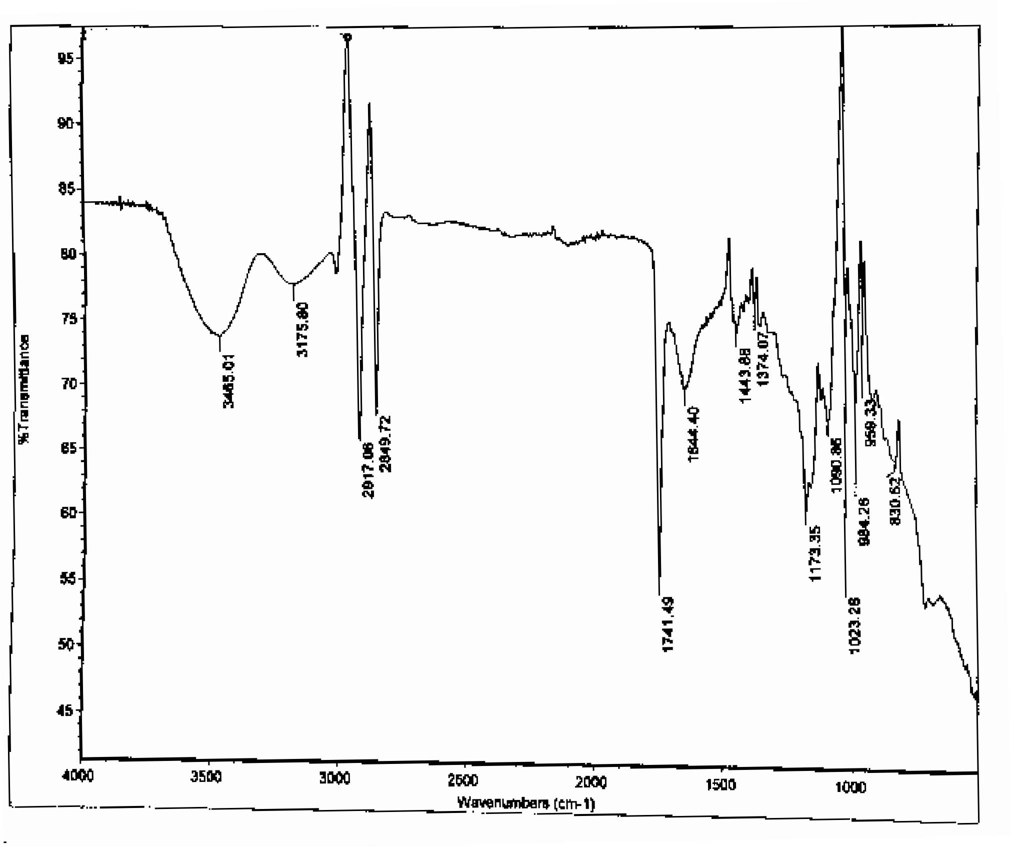
%Transmittance
Wavenumbers (cm-1)
3465.01
3175.80
2917.06
2849.72
1741.49
1644.40
1443.88
1374.07
1173.35
1090.86
1023.28
984.28
959.33
830.52

d)

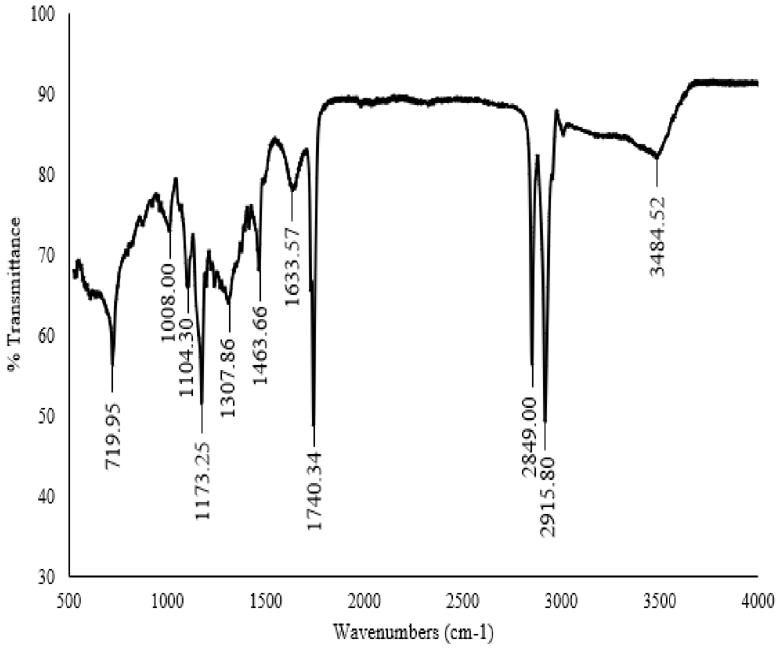
% Transmittance
Wavenumbers (cm-1)
719.95
1008.00
1104.30
1173.25
1307.86
1463.66
1633.57
1740.34
2849.00
2915.80
3484.52

e)

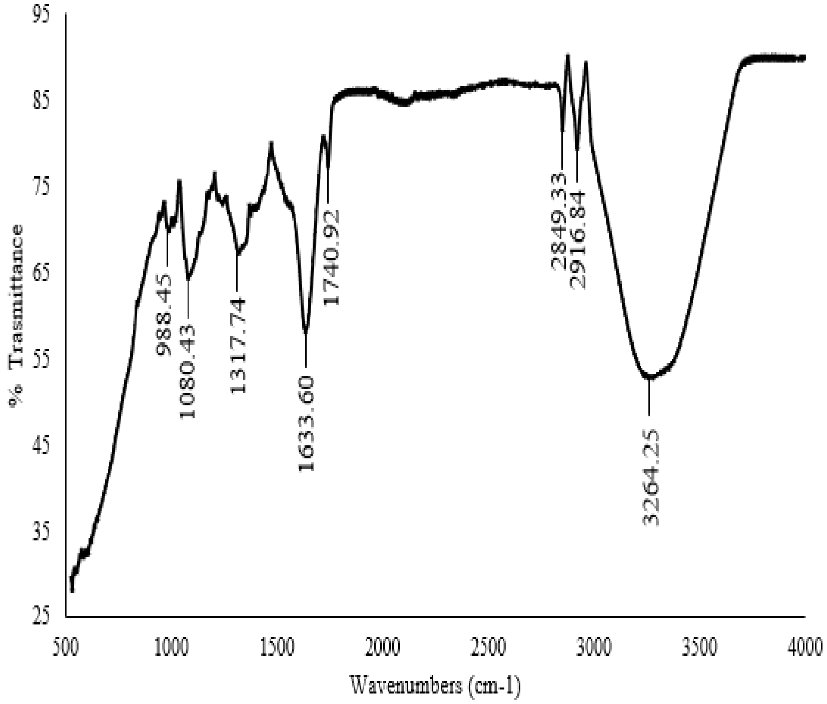
95
85
75
65
55
45
35
25
500
1000
1500
2000
2500
3000
3500
4000
% Trasmittance
Wavenumbers (cm-1)
988.45
1080.43
1317.74
1633.60
1740.92
2849.33
2916.84
3264.25

f)

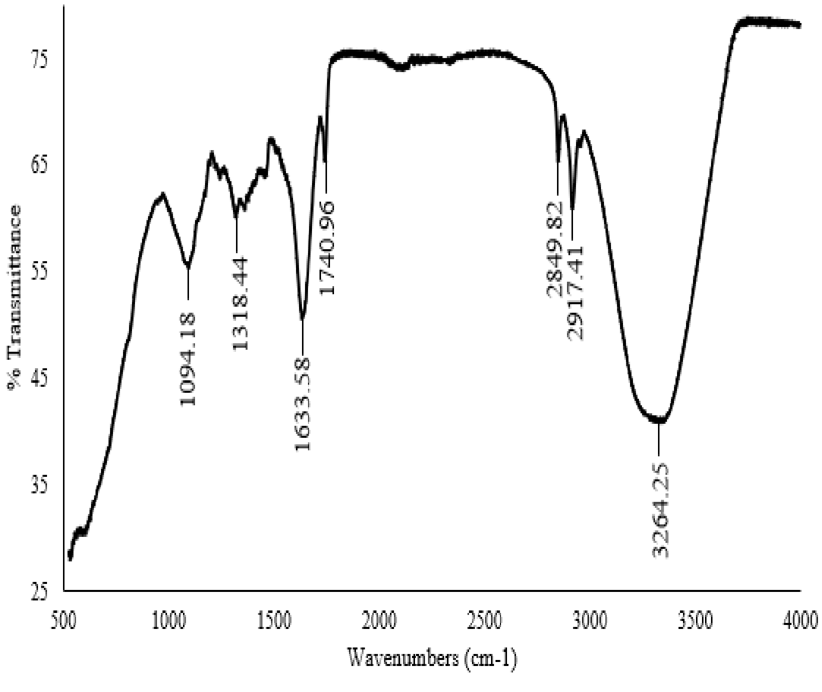
75
65
55
45
35
25
500
1000
1500
2000
2500
3000
3500
4000
% Transmittance
Wavenumbers (cm-1)
1094.18
1318.44
1633.58
1740.96
2849.82
2917.41
3264.25

g)

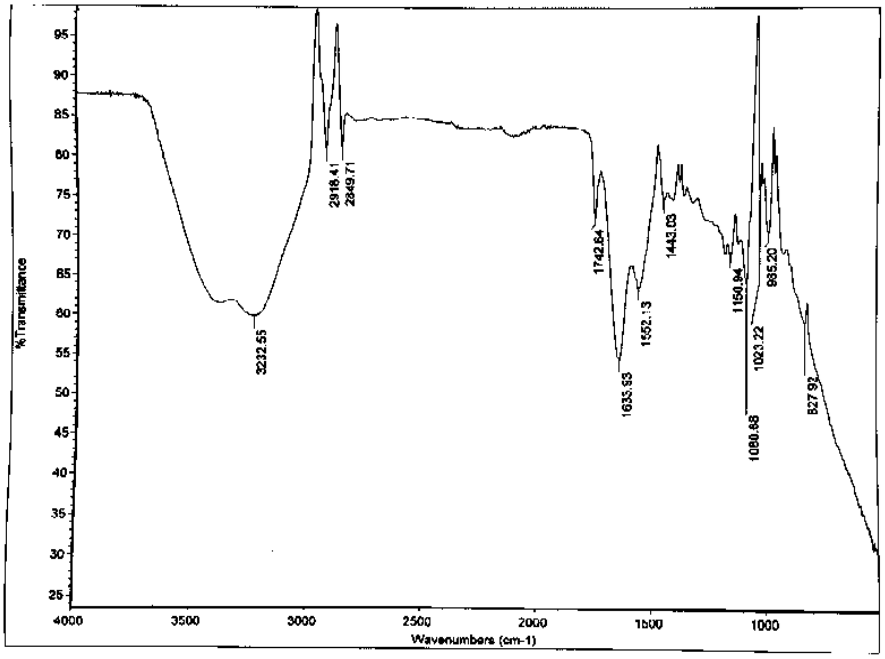

h)

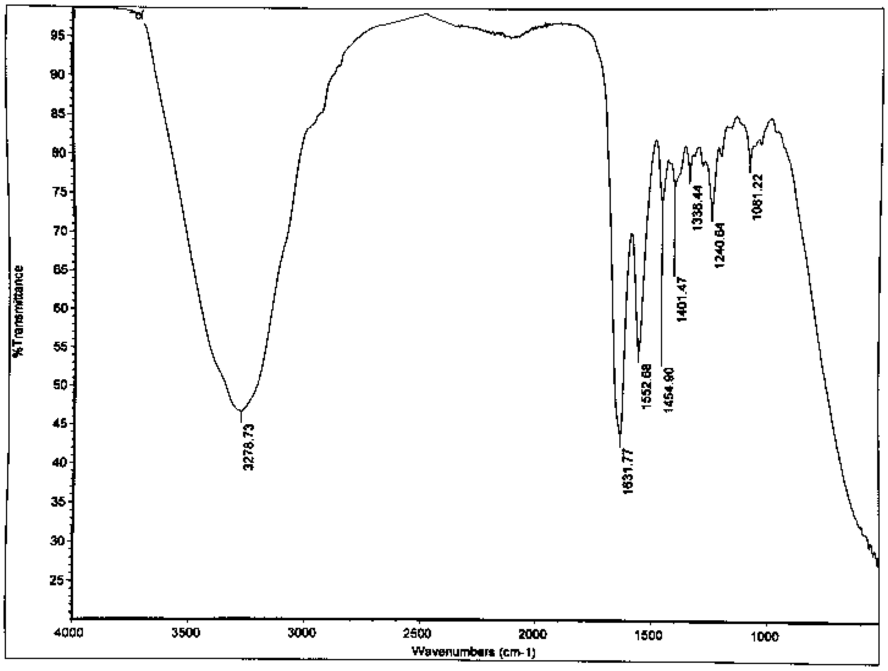

Figure S1. FT-IR Spectrums of food samples: (a) Kausar Ghee; (b) Mujahid Ghee; (c) Blue Band Margarine; (d) Belle Margarine; (e) Chibo UHT Dairy Cream; (f) Dubala Dairy Cream; (g) Cheese; (h) animal fat.

a)

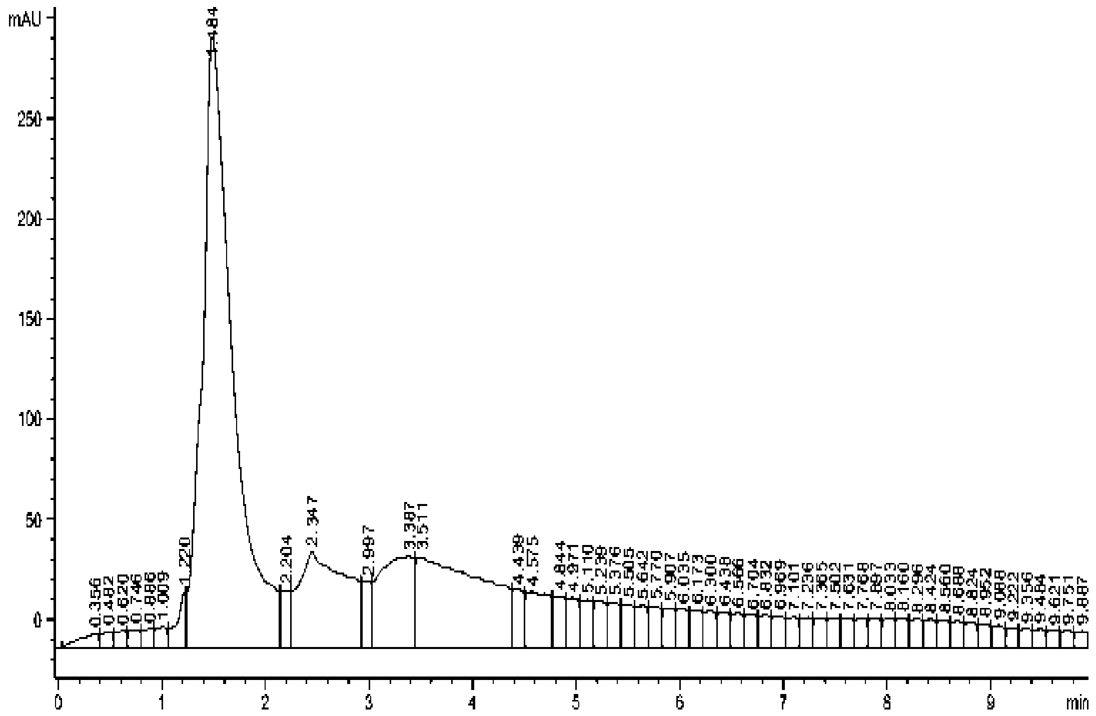
mAU
250
200
150
100
50
0
0
1
2
3
4
5
6
7
8
9
min
2.347
3.387
3.511
1.220
2.204
2.997
4.439
4.575

b)

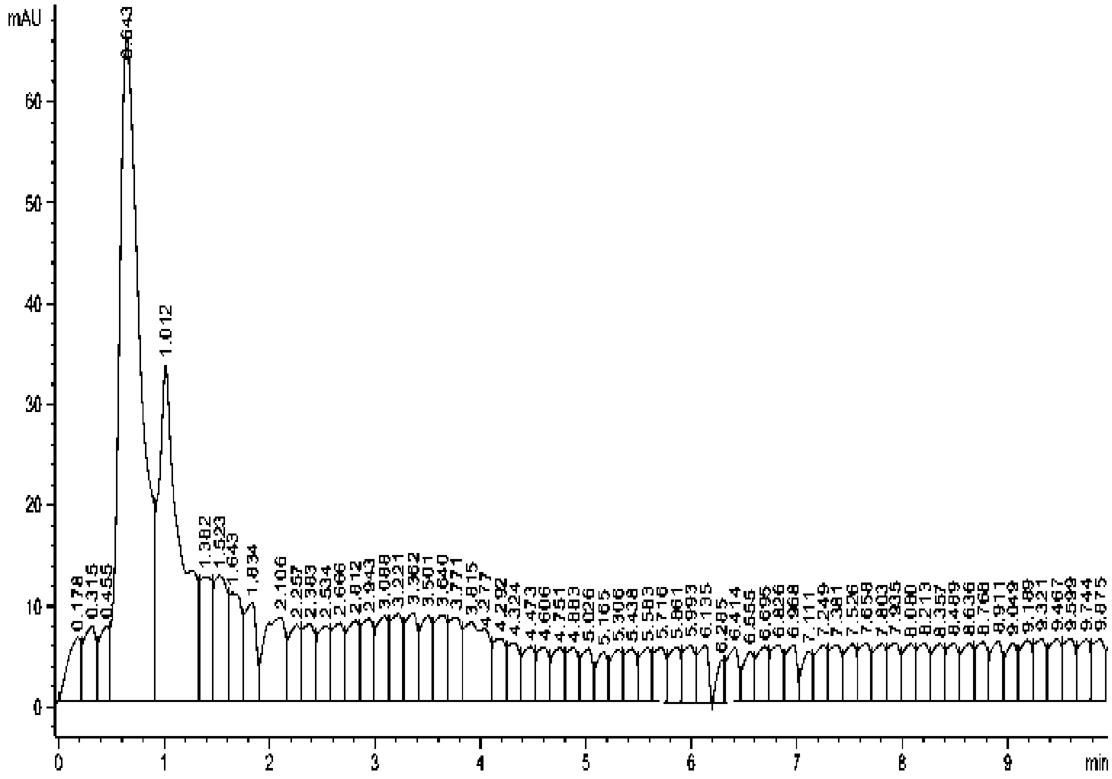
mAU
60
50
40
30
20
10
0
0
1
2
3
4
5
6
7
8
9
min
0.643
1.012
1.382
1.523
1.643
1.834
2.106

c)

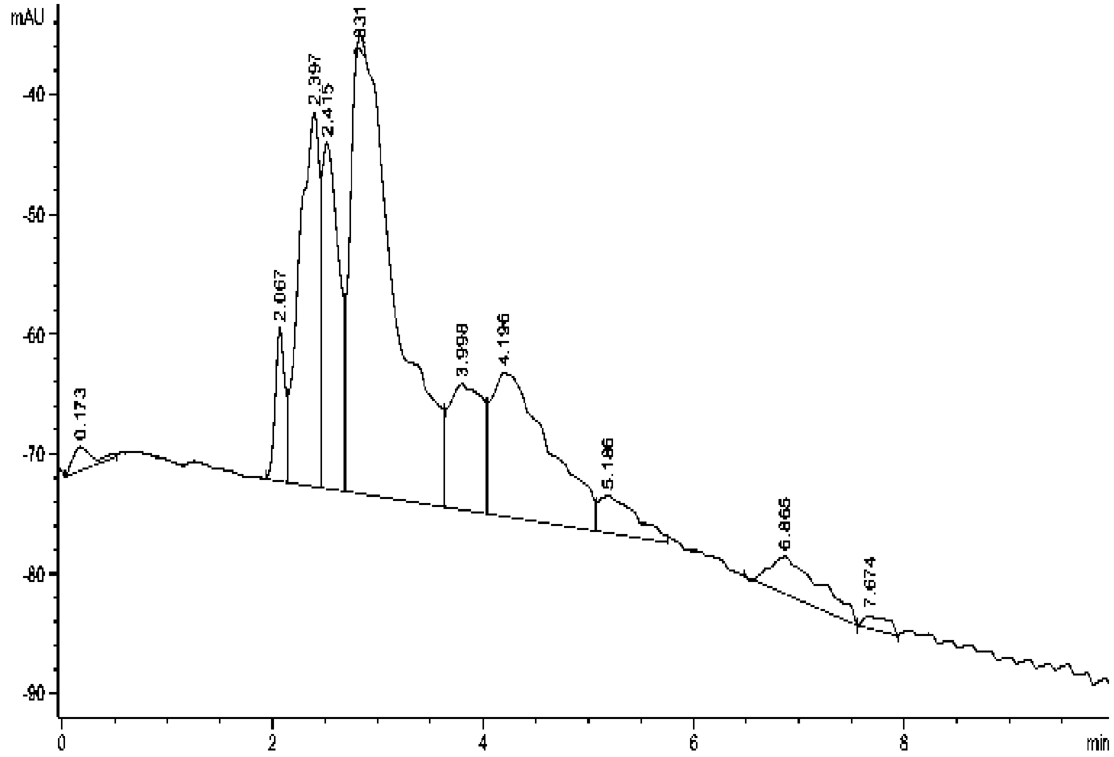
mAU
-40
-50
-60
-70
-80
-90
0
2
4
6
8
min
0.173
2.067
2.397
2.415
2.831
3.998
4.196
5.186
6.865
7.674

d)

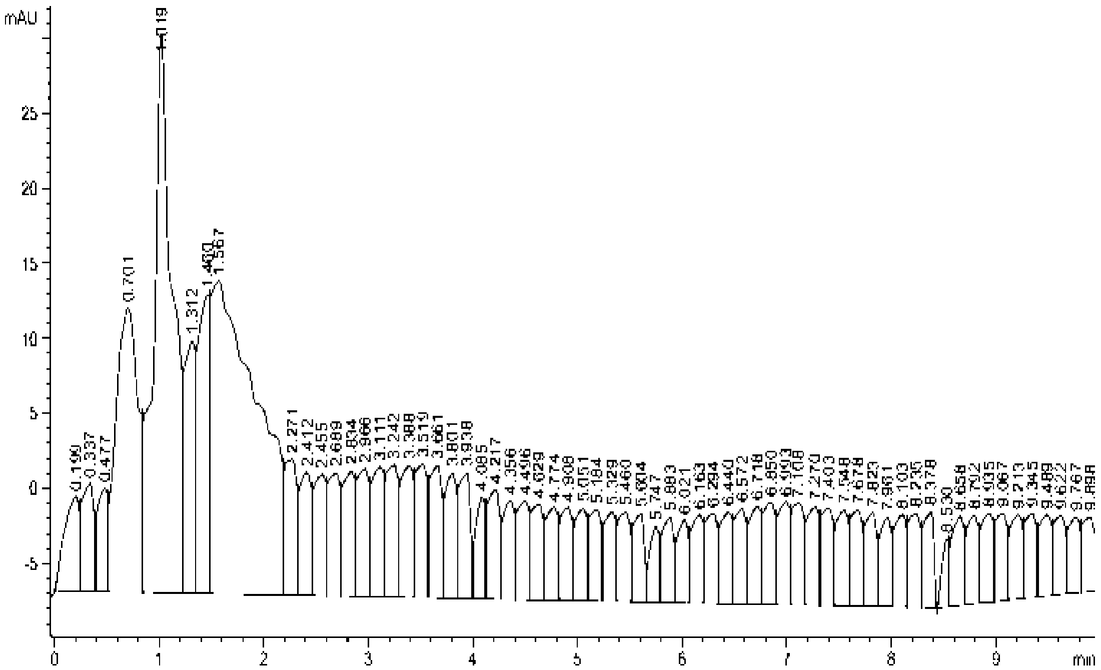
mAU
25
20
15
10
5
0
-5
0
1
2
3
4
5
6
7
8
9
min
0.199
0.337
0.477
0.701
1.019
1.312
1.400
1.567
2.271
2.412
2.455
2.689
2.834
2.966
3.111
3.242
3.388
3.519
3.661
3.801
3.938
4.085
4.217
4.356
4.496
4.629
4.774
4.908
5.051
5.184
5.329
5.460
5.604
5.747
5.883
6.021
6.163
6.294
6.440
6.572
6.718
6.850
6.993
7.108
7.270
7.403
7.548
7.678
7.823
7.961
8.103
8.235
8.378
8.530
8.658
8.792
8.935
9.067
9.213
9.345
9.489
9.622
9.767
9.898

e)

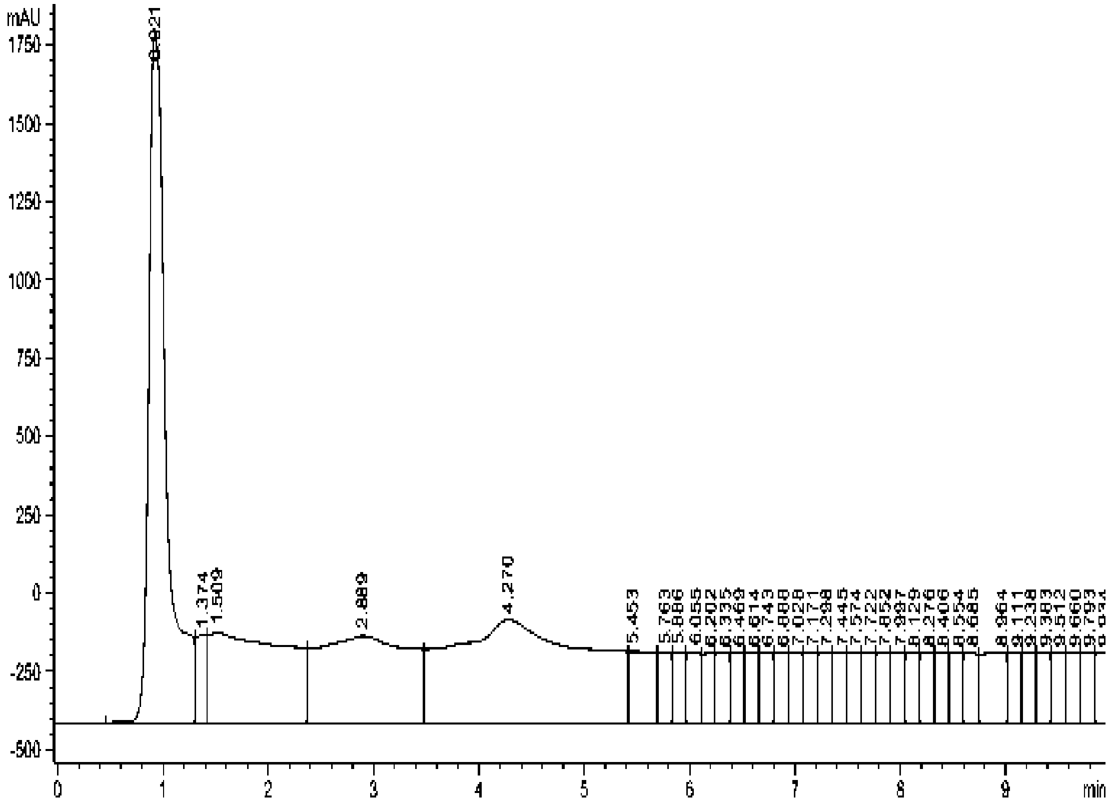

Figure S2. HPLC analysis of serum: (a) control group; (b) oleic acid group; (c) elaidic acid group; (d) Mujahid Ghee group; (e) Kausar Ghee group.

a)

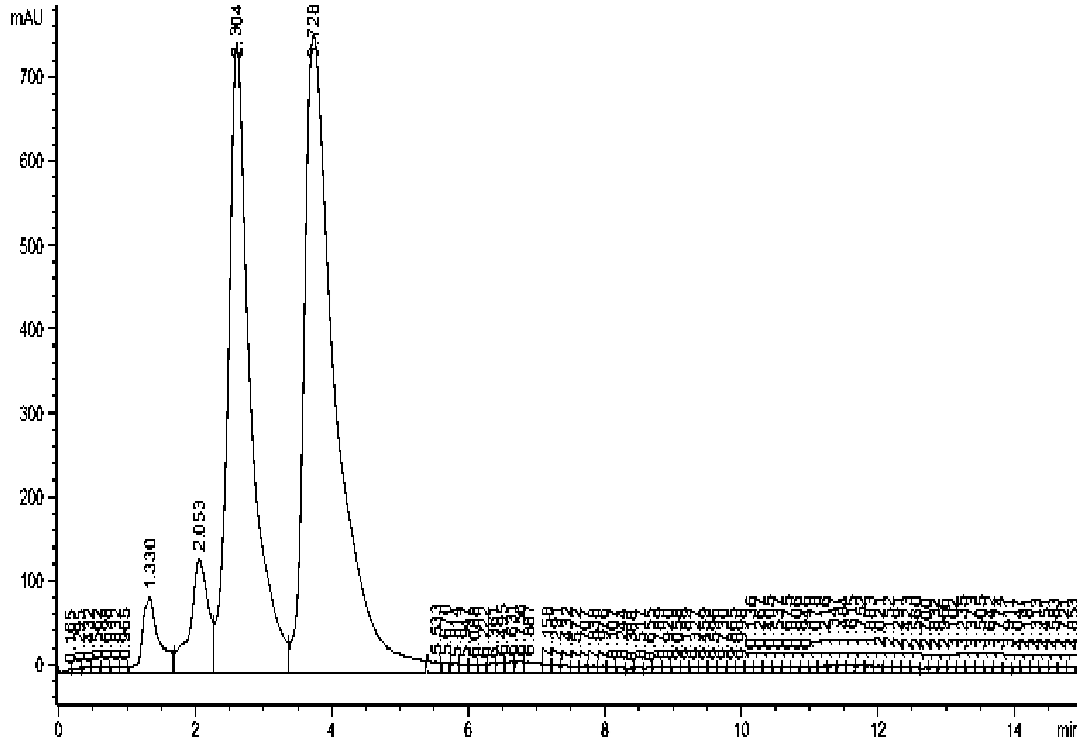
mAU
700
600
500
400
300
200
100
0
0
2
4
6
8
10
12
14
min
1.330
2.053
3.728

b)

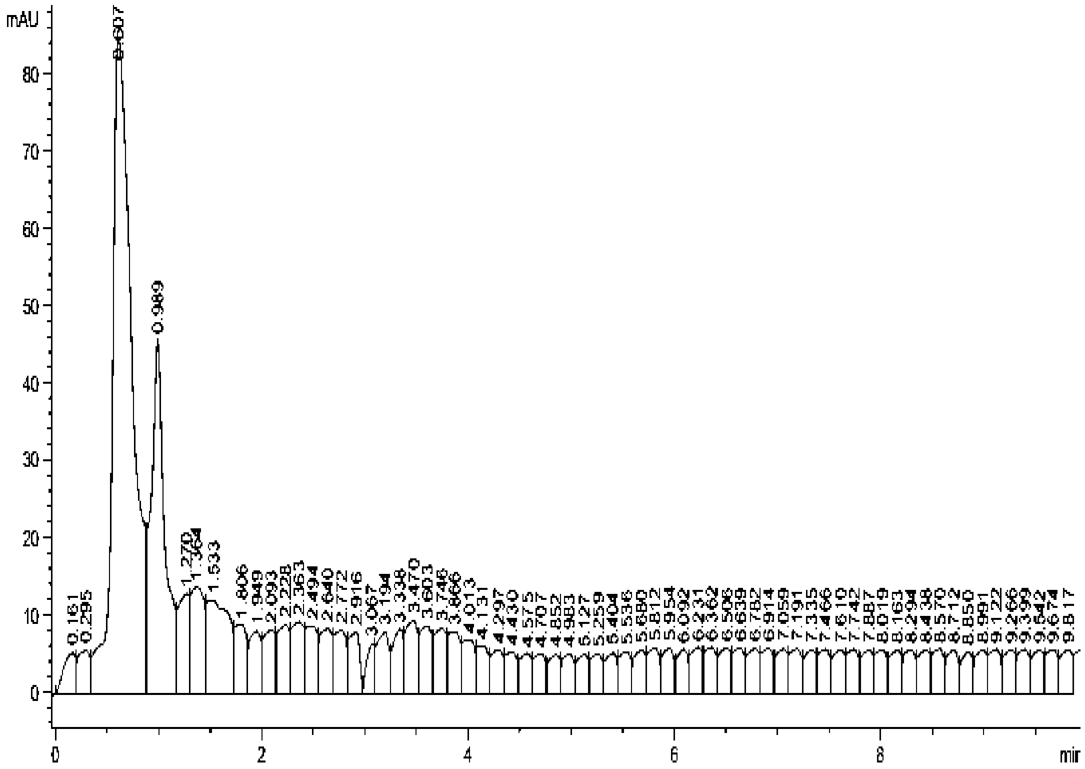
mAU
80
70
60
50
40
30
20
10
0
0
2
4
6
8
min
0.161
0.295
0.989
1.270
1.533
1.806
1.949
2.093
2.228
2.363
2.494
2.640
2.772
2.916
3.067
3.194
3.338
3.470
3.603
3.746
3.866
4.013
4.131
4.297
4.430
4.575
4.707
4.852
4.983
5.127
5.259
5.404
5.536
5.680
5.812
5.954
6.092
6.231
6.362
6.506
6.639
6.782
6.914
7.059
7.191
7.335
7.466
7.610
7.742
7.887
8.019
8.163
8.294
8.438
8.570
8.712
8.850
8.991
9.122
9.266
9.399
9.542
9.674
9.817

c)

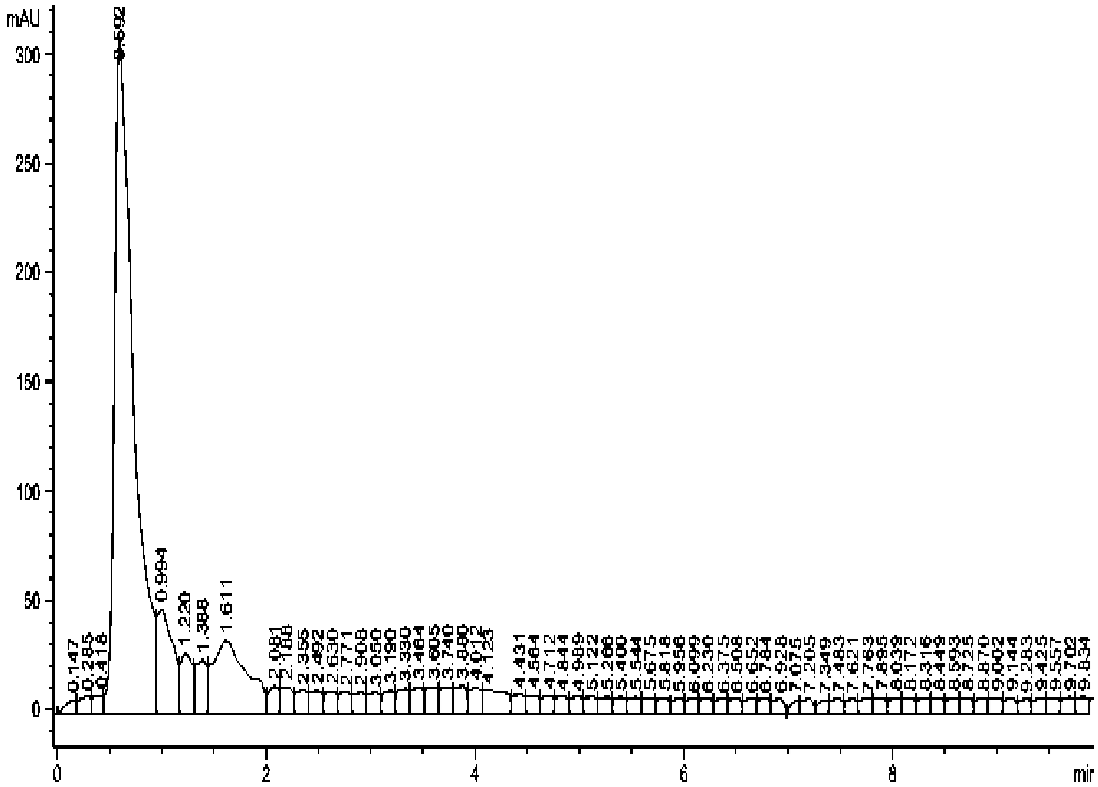

d)

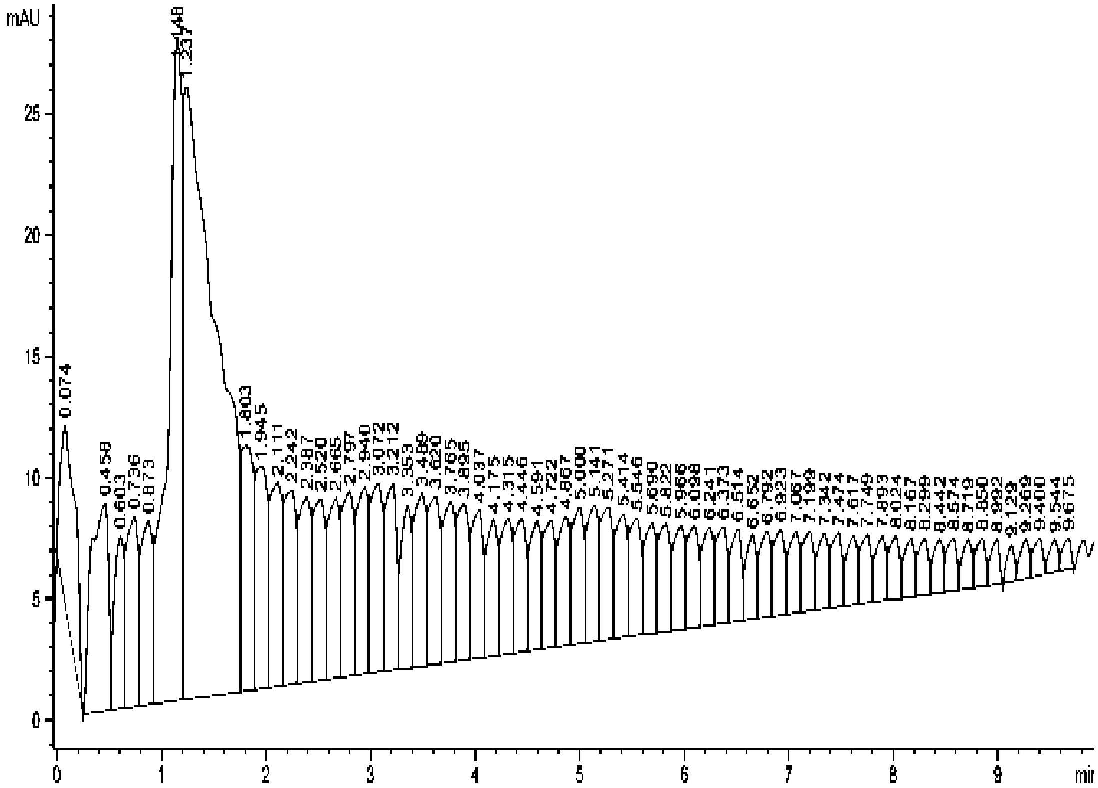

e)

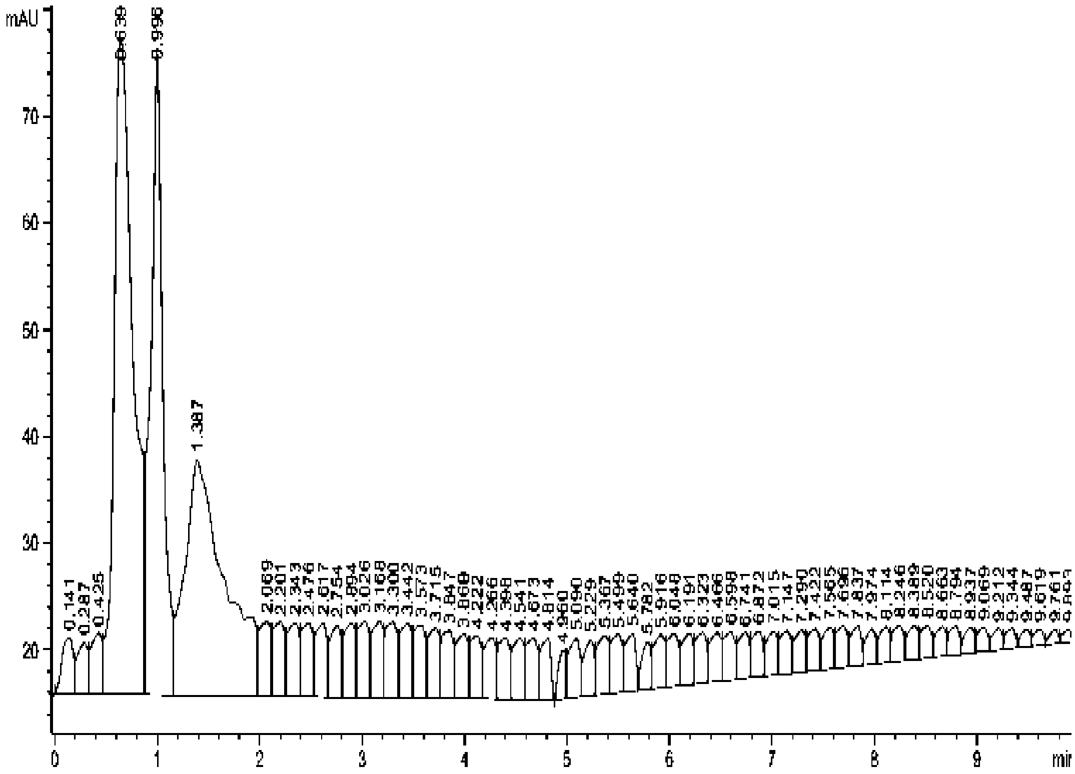

Figure S3. HPLC Results of heart tissue: (a) control group; (b) oleic acid group; (c) elaidic acid group; (d) Mujahid Ghee group; (e) Kausar Ghee group.

a)

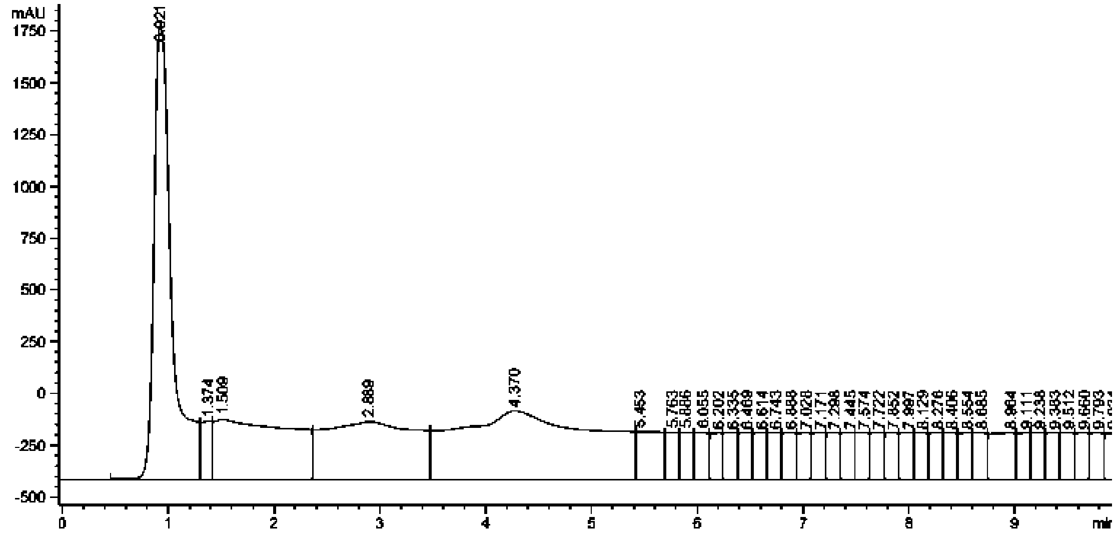
mAU
1750
1500
1250
1000
750
500
250
0
-250
-500
0
1
2
3
4
5
6
7
8
9
min
0.921
1.374
1.509
2.889
4.370
5.453

b)

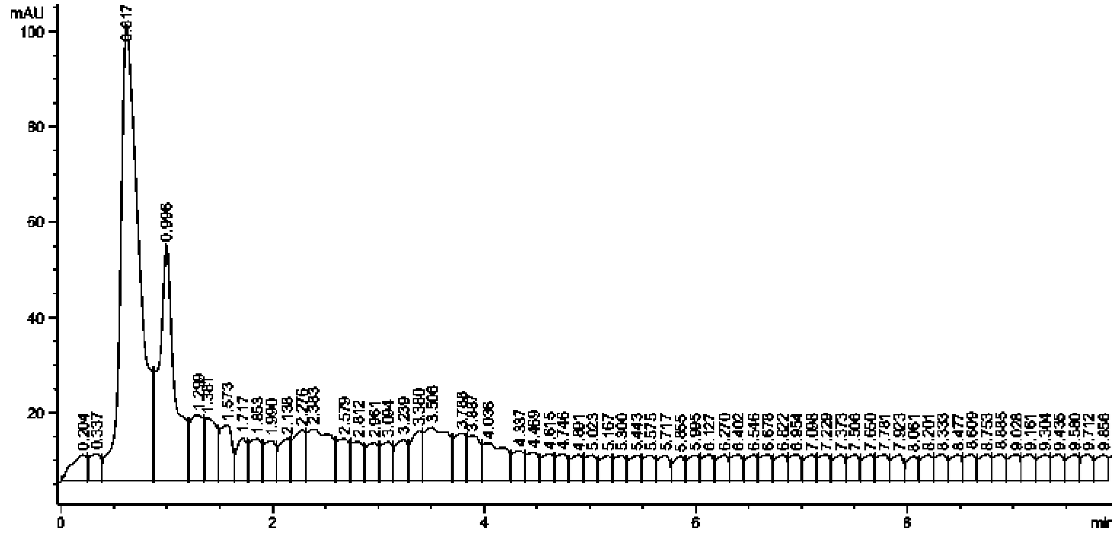
mAU
100
80
60
40
20
0
2
4
6
8
min
0.617
0.996

c)

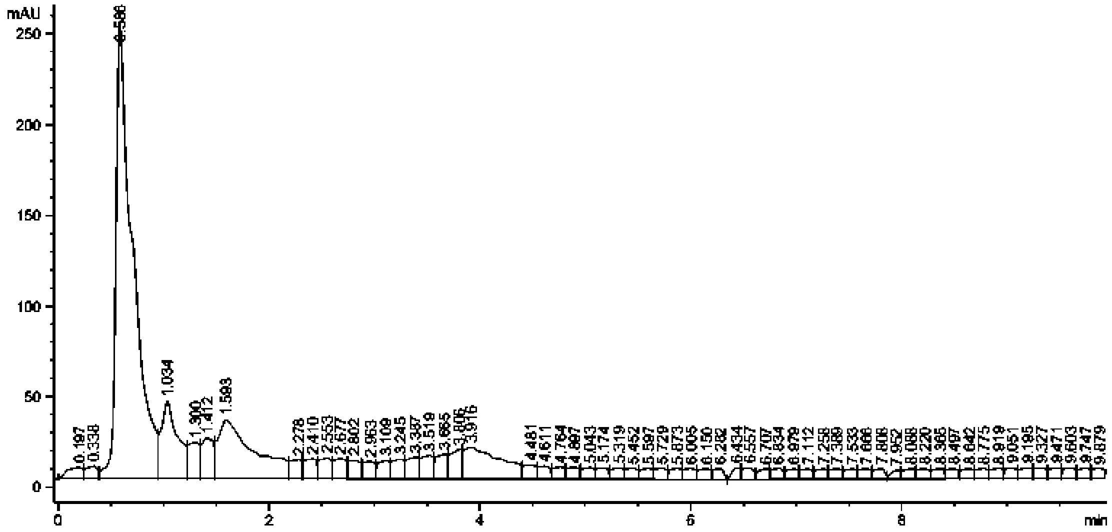

mAU
250
200
150
100
50
0
0
2
4
6
8
min
1.034
1.593

d)

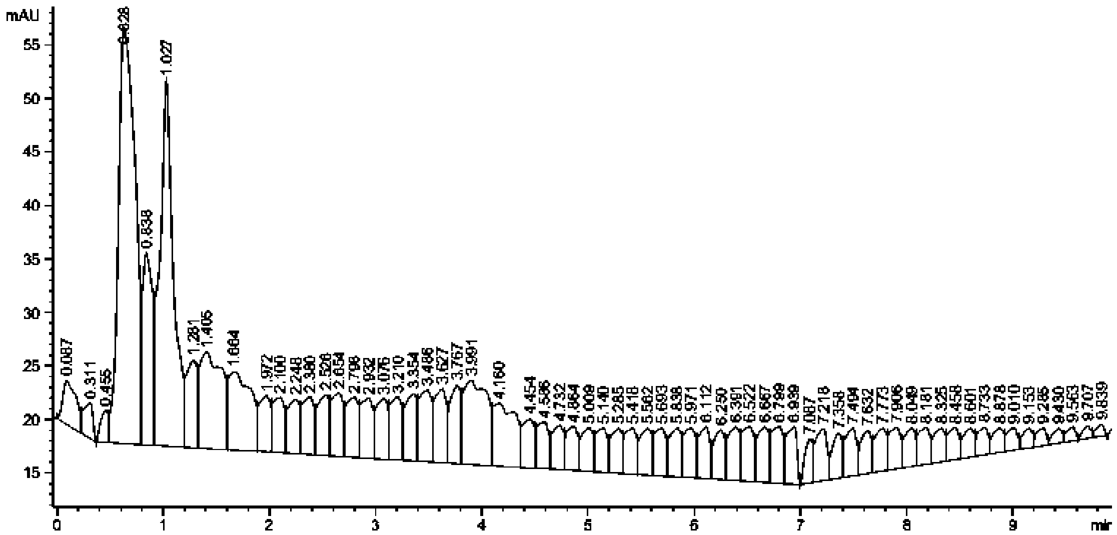

mAU
55
50
45
40
35
30
25
20
15
0
1
2
3
4
5
6
7
8
9
1.027
0.838
1.405
1.664

e)

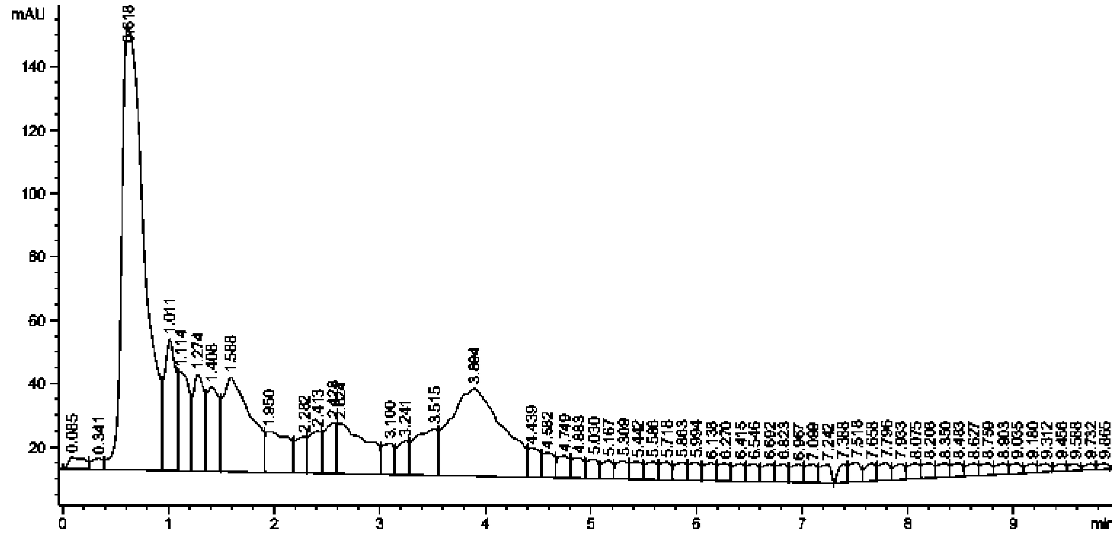

Figure S4. HPLC Results of liver tissue: (a) control group; (b) oleic acid group; (c) elaidic acid group; (d) Mujahid Ghee group; (e) Kausar Ghee group.

a)

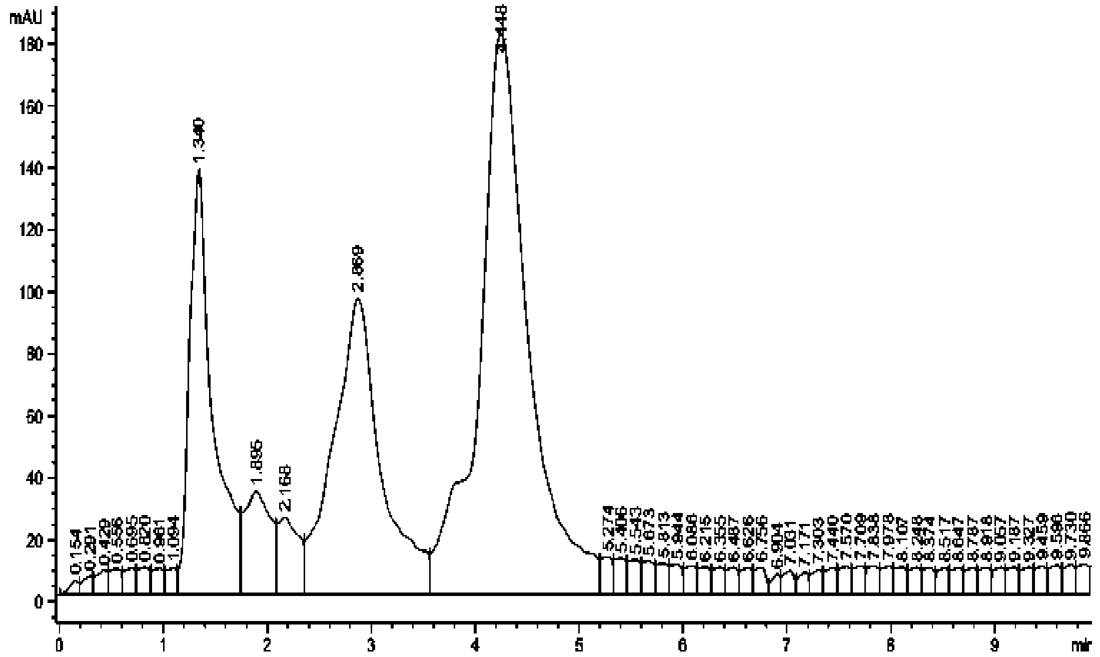

b)

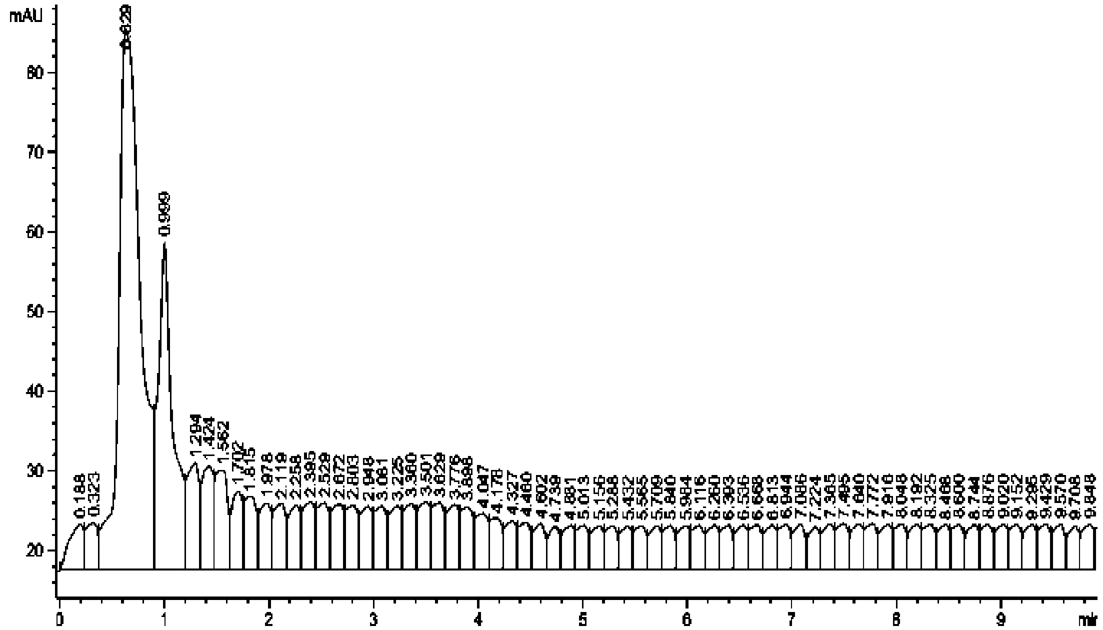

c)

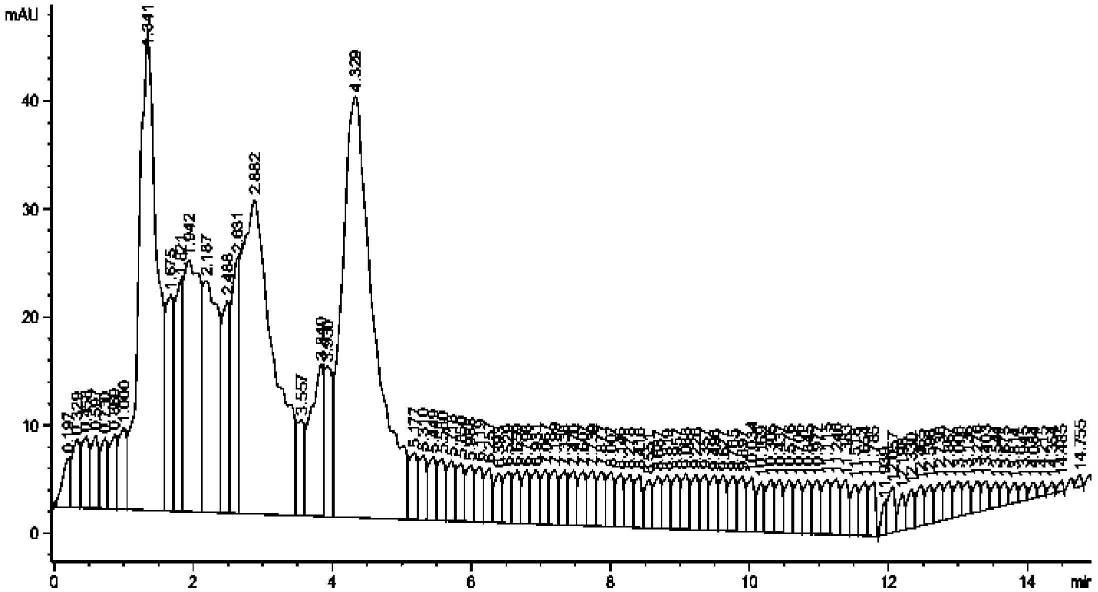

mAU
40
30
20
10
0
1.341
2.882
4.329
14.755
0
2
4
6
8
10
12
14

d)

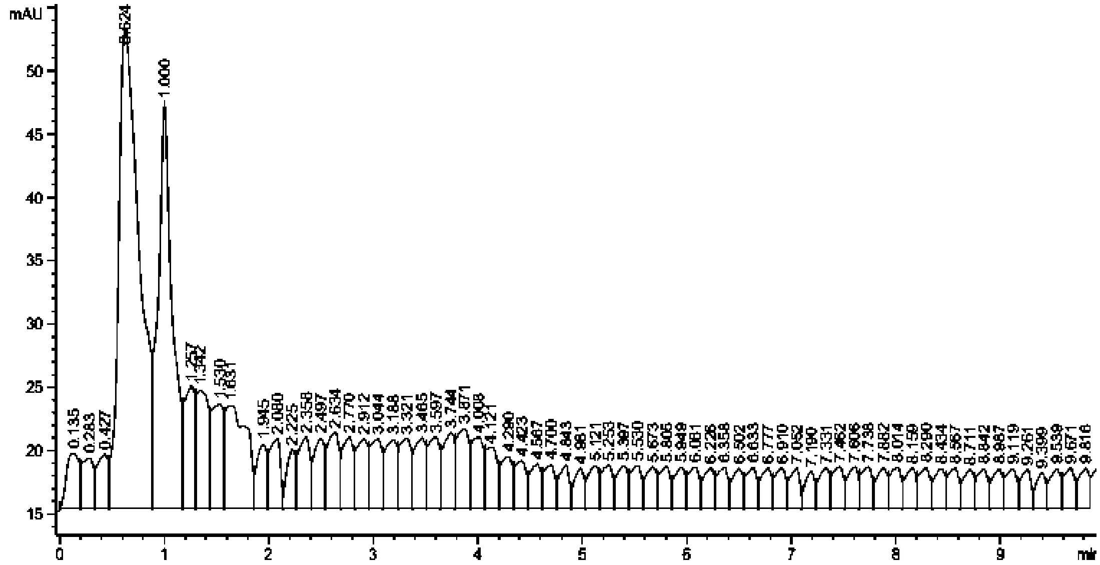

mAU
50
45
40
35
30
25
20
15
1.000
0
1
2
3
4
5
6
7
8
9

e)

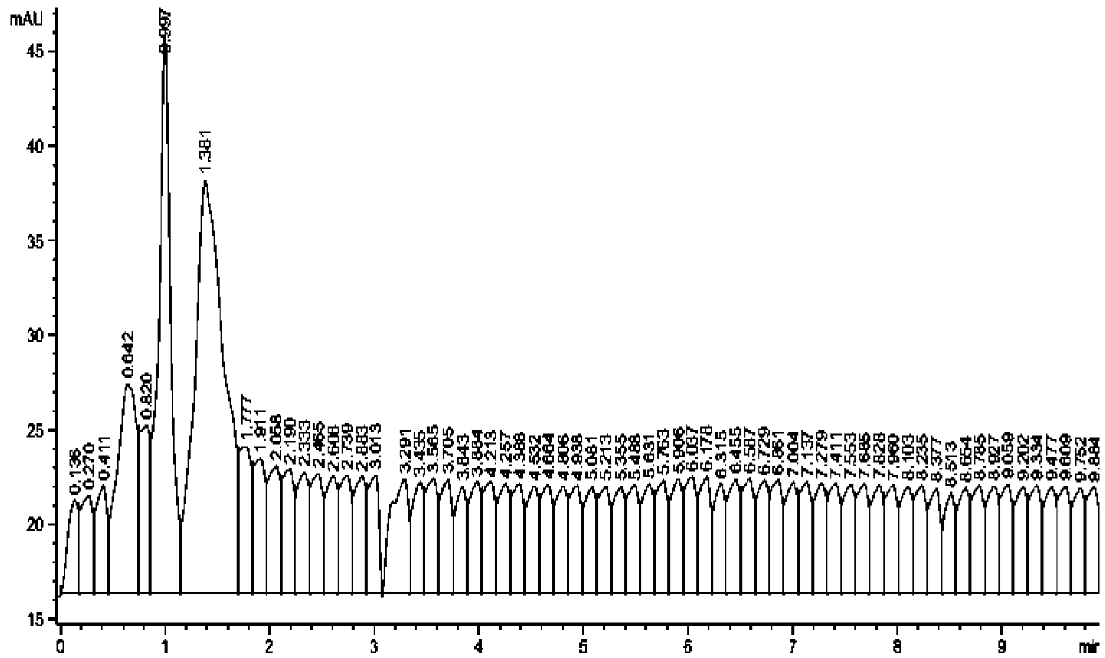

Figure S5. HPLC Results of kidney tissue: (a) control group; (b) oleic acid group; (c) elaidic acid group; (d) Mujahid Ghee group; (e) Kausar Ghee group.
